# Development of an EORTC questionnaire measuring instrumental activities of daily living (IADL) in patients with brain tumours: phase I–III

**DOI:** 10.1007/s11136-020-02738-5

**Published:** 2021-01-26

**Authors:** Quirien Oort, Linda Dirven, Sietske A. M. Sikkes, Neil Aaronson, Florien Boele, Christine Brannan, Jonas Egeter, Robin Grant, Martin Klein, Irene Lips, Yoshitaka Narita, Hitomi Sato, Monika Sztankay, Günther Stockhammer, Andrea Talacchi, Bernard M. J. Uitdehaag, Jaap C. Reijneveld, Martin J. B. Taphoorn, on behalf of the EORTC Quality of Life Group

**Affiliations:** 1 Department of Neurology and Brain Tumor Center Amsterdam, Amsterdam UMC, Vrije Universiteit Amsterdam, PO BOX 7057, 1007 MB Amsterdam, The Netherlands; 2 Department of Neurology, Leiden University Medical Center, Leiden, The Netherlands; 3 Department of Neurology, Haaglanden Medical Center, The Hague, The Netherlands; 4 Department of Epidemiology and Biostatistics, Amsterdam UMC, Vrije Universiteit Amsterdam, Amsterdam, The Netherlands; 5 Alzheimer Center, Amsterdam UMC, Vrije Universiteit Amsterdam, Amsterdam, The Netherlands; 6 Department of Clinical Developmental & Clinical Neuropsychology, Faculty of Behavioural and Movement Sciences (FGB), Vrije Universiteit Amsterdam, Amsterdam, The Netherlands; 7 Division of Psychosocial Research and Epidemiology, The Netherlands Cancer Institute, Amsterdam, The Netherlands; 8 Leeds Institute of Medical Research, St James’s University Hospital, Leeds, LS9 7TF UK; 9 Faculty of Medicine and Health, Leeds Institute of Health Sciences, University of Leeds, Leeds, LS2 9JT UK; 10 East & North Hertfordshire NHS Trust Incorporating Mount Vernon Cancer Centre, Northwood, UK; 11 Department for Psychiatry, Psychotherapy and Psychosomatics, University Hospital of Psychiatry II, Medical University of Innsbruck, Innsbruck, Austria; 12 Department of Clinical Neurosciences, Western General Hospital, Edinburgh, UK; 13 Department of Medical Psychology, Amsterdam UMC, Vrije Universiteit Amsterdam, Amsterdam, The Netherlands; 14 Department of Radiation Oncology, Leiden University Medical Center, Leiden, The Netherlands; 15 Department of Neurosurgery and Neuro-Oncology, National Cancer Center, Tokyo, Japan; 16 Department of Nursing, Teikyo Heisei University, Tokyo, Japan; 17 Department of Neurology, Innsbruck Medical University, Innsbruck, Austria; 18 Department of Neurosurgery, Azienda Ospedaliera San Giovanni Addolorata, Rome, Italy; 19 Department of Neurology, Stichting Epilepsie Instellingen Nederland (SEIN), Heemstede, The Netherlands

**Keywords:** Daily functioning, Instrumental activities of daily living, IADL, Brain tumour, Questionnaire

## Abstract

**Purpose:**

Being able to function independently in society is an important aspect of quality of life. This ability goes beyond self-care, requires higher order cognitive functioning, and is typically measured with instrumental activities of daily living (IADL) questionnaires. Cognitive deficits are frequently observed in brain tumour patients, however, IADL is almost never assessed because no valid and reliable IADL measure is available for this patient group. Therefore, this measure is currently being developed.

**Methods:**

This international multicentre study followed European Organisation for Research and Treatment of Cancer (EORTC) Quality of Life Group module development guidelines. Three out of four phases are completed: phases (I) generation of items, (II) construction of the item list, and (III) pre-testing. This paper reports the item selection procedures and preliminary psychometric properties of the questionnaire. Brain tumour patients (gliomas and brain metastases), their informal caregivers, and health care professionals (HCPs) were included.

**Results:**

Phase I (*n* = 44 patient-proxy dyads and 26 HCPs) generated 59 relevant and important activities. In phase II, the activities were converted into items. In phase III (*n* = 85 dyads), the 59 items were pre-tested. Item selection procedures resulted in 32 items. Exploratory factor analysis revealed a preliminary dimensional structure consisting of five scales with acceptable to excellent internal consistency (*α* = 0.73–0.94) and two single items. For three scales, patients with cognitive impairments had significantly more IADL problems than patients without impairments.

**Conclusion:**

A phase IV validation study is needed to confirm the psychometric properties of the EORTC IADL-BN32 questionnaire in a larger international sample.

**Supplementary Information:**

The online version contains supplementary material available at 10.1007/s11136-020-02738-5.

## Introduction

Brain tumour patients may suffer from various physical, neurological, and neurocognitive impairments. These impairments can have a substantial negative impact on a patient’s ability to function in everyday life. Everyday life, or daily functioning, can be measured on two levels: basic activities of daily living (BADL), which are related to self-maintenance (e.g. eating or dressing), and instrumental activities of daily living (IADL), which are related to autonomous functioning in society (e.g. household activities or using a computer) [1]. IADL rely more heavily on higher order cognitive functioning. Deterioration of cognition is associated with worse performance on IADL in the general [2] and elderly population [1], and in patients prone to cognitive impairments, such as dementia [3].

Despite the fact that preserving the ability to function independently as long as possible is particularly important to brain tumour patients due to the incurable nature of the disease, and cognitive decline is frequently observed in this patient group [4], IADL is almost never measured in brain tumour trials or used in clinical practice because no reliable and validated measures are available for this patient group [5].

One study evaluated the applicability of a reliable and valid IADL questionnaire developed for dementia patients (i.e. Amsterdam IADL Questionnaire^©^ (A-IADL-Q)) [3, 6–8], for the brain tumour population [9], since both patient groups experience similar cognitive problems [10, 11]. However, this instrument did not appear to be entirely relevant to brain tumour patients, warranting a brain tumour-specific IADL questionnaire.

The general consensus is that patients are the best source to rate their functioning and well-being [12]. The A-IADL-Q, however, was developed as a proxy-based questionnaire, as it was hypothesized that cognitive deficits could potentially limit the dementia patients’ ability to rate their own level over daily functioning. Brain tumour patients could be similarly limited in their ability to rate their level of functioning [13]. Therefore, both a patient-based and proxy-based version are being developed, analysed, and compared, after which it can be decided what version is most appropriate in the brain tumour setting.

The aim of this study is to develop a reliable and valid IADL questionnaire that can be implemented in both brain tumour trials and clinical practice. Here, the first three phases of the development of the questionnaire are described.

## Methods

### Study design

**Fig. 1:**
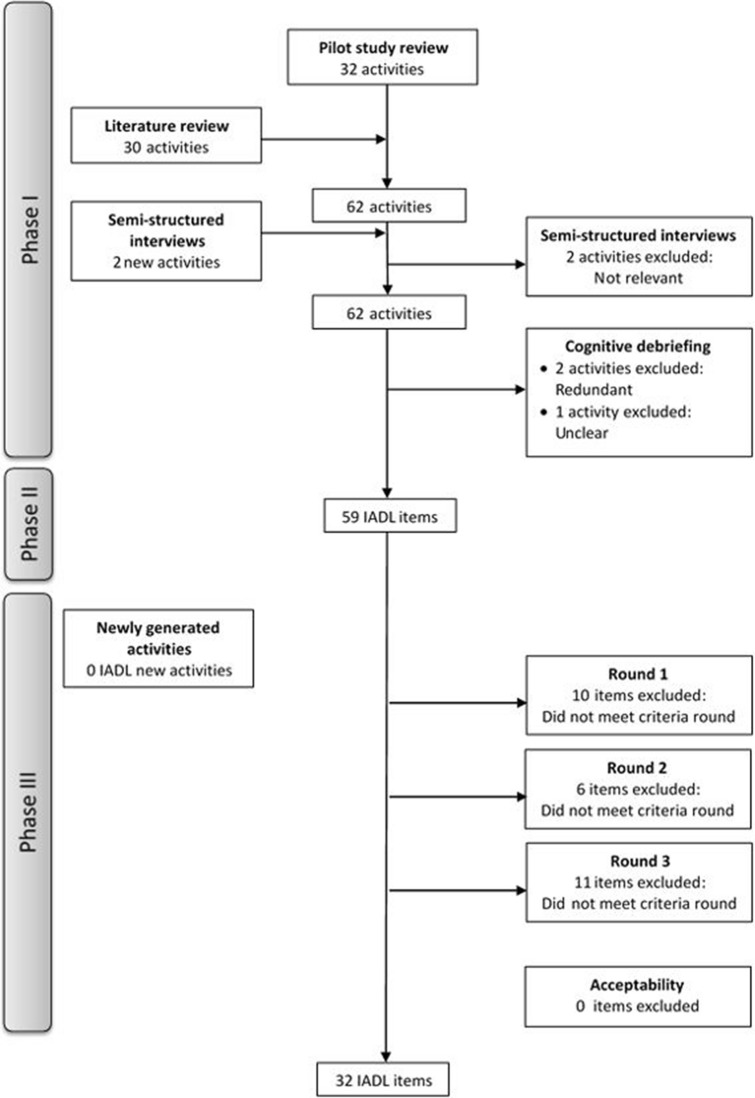
Flowchart item selection phases I-III

The European Organisation for Research and Treatment of Cancer (EORTC) Quality of Life Group (QLG) guidelines for module development [14] were followed, consisting of four phases: (I) Generation of items, (II) Construction of the item list, (III) Pre-testing, and (IV) Field testing. This paper reports the results of phases I–III of the developmental process (for more details, see Supplemental File 1; Fig. 1).

### Study population

The study population consisted of patients with a primary or metastatic brain tumour, their informal caregivers as proxies, and centre-affiliated health care professionals (HCPs) in the field of neuro-oncology. Patients were eligible if they had either a histologically confirmed glioma (based on WHO 2007 criteria) or brain metastases and a histologically confirmed primary tumour. Further inclusion criteria were age (≥ 18 years) and contact frequency between the patient and their proxy (daily or weekly) to ensure a reliable assessment of the patient’s daily functioning. The exclusion criterion was an insufficient understanding of the official language of the country of residence to complete study procedures.

Participant recruitment for phase I took part consecutively in both academic and non-academic outpatient clinics in the Netherlands, Italy, and the United Kingdom and for phase III in three European regions, namely Northern Europe (The Netherlands and Austria), Southern Europe (Italy), an English-speaking region (the United Kingdom, i.e. England and Scotland), and a non-European country (Japan). Patients meeting the in- and exclusion criteria, who were also determined to be physically (i.e. performance status) and mentally (i.e. able to consent to research and have the mental capacity to complete the study procedures) fit to participate by their treating physicians, were approached to participate in the study. Following EORTC QLG module development guidelines, patient recruitment targeted an even distribution across the relevant variables, i.e. tumour type (high-grade glioma (HGG)/low-grade glioma (LGG)/1–3 brain metastases/ > 3 brain metastases) in phase I and, additionally, the presence of cognitive deficits (present/not present) in phase III. Both patient and proxies provided written informed consent before participation.

### Phases I & II

Phase I aimed to compile an extensive list of IADL relevant to brain tumour patients. Five sources of information were used: (1) the relevant A-IADL-Q [7] activities from a previous study (referred to as ‘pilot study’) in glioma patients [9], and in accordance with EORTC QLG module development guidelines, (2) the literature, (3) the EORTC Item library [15], and semi-structured interviews with (4) patients and their proxies and (5) HCPs.

During the pilot study (source 1), HCPs (*N* = 6) judged if the activities from the A-IADL-Q could be considered as ‘IADL’ using the definition: ‘IADL are complex activities with little automated skills for which multiple cognitive processes are necessary [6]?’. If ≥ 2 HCPs rated an activity as not being IADL, it was excluded from further analysis. Subsequently, HCPs as well as patients (*N* = 12) and proxies (*N* = 12) had to rate if the proposed activities were ‘likely to be affected in brain tumour patients’. Activities were excluded if more than half of all participant groups rated the activity as not affected. Finally, patients, proxies, and HCPs evaluated if the activities from the A-IADL-Q were ‘clearly formulated’. In this case, if an activity was rated unclear by > 4 HCPs or > 10 patients or > 10 proxies, the formulation was reviewed and the item rephrased.

From a literature review of the electronic databases PubMed, Embase, Cochrane, PsycINFO and CINAHL conducted up to April 2017 (source 2) and a review of the items in the EORTC Item Library (source 3), additional potentially relevant IADL were extracted.

Subsequently, a new group of patients (*N* = 28), proxies (*N* = 27) and HCPs (*N* = 18) participated in the semi-structured interviews. Each IADL activity generated from sources 1–3 was rated on both relevance and importance (4‐point Likert‐scale) by patients and proxies, and missing IADL were identified. HCPs also rated the activities on relevance, and in addition provided a top 10 of the most important IADL activities. Items with an average score of < 2.0 on both relevance and importance by either the patients or proxies, or with ≥ 6 HCPs rating them as not relevant, were excluded, except if they were in ≥ 2 HCPs top 10 most important activities.

Finally, another group of patients (*N* = 2), proxies (*N* = 2), and HCPs (*N* = 2) cognitively debriefed the remaining items resulting from sources 1–5 item generation and selection process, and evaluated the appropriateness of the wording of the items, and any potential redundancies (for more details, see Supplemental File 1).

In Phase II, the activities were converted into questionnaire items and a preliminary IADL questionnaire was constructed. Both a patient‐based and proxy‐based version of the IADL questionnaire were constructed and subsequently translated by the EORTC Translation Unit [16] into the languages of the countries participating in phase III (English, Dutch, Italian, German, and Japanese). The proxy-based version consists of the same items but refers to the patient (e.g. ‘has he/she had difficulties’).

### Phase III

Phase III aimed to pre-test the preliminary IADL questionnaire by means of semi-structured interviews and neuropsychological testing. The interview consisted of four parts: (1) completion of the IADL questionnaire (4‐point Likert‐scale [‘not at all’- ‘very much’], and not applicable), (2) rating each activity on relevance and importance (4‐point Likert‐scale) and acceptability (e.g. not too difficult/confusing/annoying/upsetting), (3) identification of the 10 most important activities, and (4) identification of missing activities. For the known-group comparison analysis, patients were classified as with or without cognitive impairments using the standard neuropsychological test battery used in EORTC trials which comprises three objective tests (six outcomes) and a subjective cognitive complaints questionnaire (one outcome) (for more details, see Supplemental File 1). Patients scoring < 2 standard deviations (SD) below the mean of the official norm scores on ≥ 2 out of 7 outcomes were classified as cognitively impaired.

#### Item selection

The item selection decision rules in phase III were based on EORTC QLG module development guidelines ensuring content validity. Cross-cultural validity was preserved by using a 3-round stepwise item selection procedure to ensure that potential skewed geographical patient population distributions would not influence item selection (see Table 1 for all decision rules).

**Table 1:** Predetermined decision rules for item inclusion, exclusion, and revision for phases I–III

Phases	Predetermined decision rules
**Phase I**	
Literature review	
*Include*	Activities that could be considered IADL
Step 1. Activities ‘pilot study’	
* Include*	32 pilot study IADL activities
*Exclude*	≥ 2 HCPs: Not considered IADL < 3 Pt/Pr/HCPs: Affected
*Revision*	≥ 2 Pt/Pr/HCPs: Unclear; rephrase or, if this was not possible, exclude
Steps 2 & 3. Semi-structured interviews	
*Include*	≥ 2 Pt/Pr/HCPs: New activity generated that could be considered IADL
*Exclude*	≥ 6 HCPs: Mean relevance & importance < 2.0 (unless ≥ 2 HCPs in top 10) or Not relevant
Step 4. Cognitive debriefing	
*Exclude*	≥ 2 Pt/Pr/HCPs: Activities are too similar (redundancy)
*Revision*	≥ 2 Pt/Pr/HCPs: Unclear; rephrase or, if this was not possible, exclude
**Phase II**	
Activities converted to items	
*Revision*	Revisions based on recommendations from the Translation Unit to be applicable in all languages
**Phase III**	
Newly generated items	
*Include*	≥2 Pt/Pr: New activity generated that could be considered IADL
Round 1: Per geographical region	
*Exclude*	Pt: Not in the top 10 most important items and < 60% score 3 or 4 on both importance & relevance and Mean score < 2.0 on difficulty with item (N/A responses excluded)
Round 2: All geographical regions	
*Exclude: (in > 1 region & unless in top 10 most important)*	Pt: < 60% score 3 or 4 on both importance & relevance or ≥ 75% ‘Not applicable’
Round 3: All geographical regions	
*Exclude: (in > 1 region & unless in top 10 most important)*	Pt: Prevalence ratio > 30% or Floor or ceiling effect scores 1&2 or 3&4 were > 20% or Compliance rate ≤ 95% or Range ≤ 2 points
Acceptability	
*Revision per region*	> 5 Pt&Pr: Rate item difficult/confusing/annoying/upsetting
*Revision all regions*	> 5% Pt&Pr (8.5 Pt&Pr): Rate item difficult/confusing/annoying/upsetting

*Pt* patients, *Pr* proxies, *HCPs* health care professionals, *IADL* instrument activities of daily living

Item retention/omission was based on patient data only. However, a sensitivity analysis was performed by repeating these steps with proxy data.

#### Statistical analysis

Descriptive statistics were used to describe the sociodemographic and clinical characteristics of the participants, as well as the quantitative data in phases I and III.

A preliminary evaluation of several psychometric properties was performed. The content validity was ensured by using multiple sources of information to identify IADL (phase I), and the subsequent evaluation of relevance, importance, acceptability, and completeness of the item list in phase III (Table 1). Cross-cultural validity was ensured by including participants from different geographical regions and the 3-round stepwise item selection procedure. After the item selection procedure, structural validity was assessed by performing an exploratory factor analysis (EFA; principal component analysis with orthogonal rotation (varimax)) due to the small sample and no a priori known scale structure. This included analysis of eigenvalues, with values > 1 considered as indication for a factor that should be remained, and a scree plot inspection to determine the number of factors to retain in the EFA. Multiple imputation techniques were used for 7% of observations because of the large number of patients (78%) responding ‘not applicable’ to one or more items. As ‘not applicable’ responses are coded as missing data, and standard EFA uses listwise deletion, this technique prevented that a limited number of patients could be included in the analysis.

The EFA resulted in single- and multi-item scales that were used in further analyses. The internal consistency of the multi-item scales was determined by calculating Cronbach’s alpha, with scores between 0.8 > *α* ≥ 0.7 classified as acceptable, 0.9 > *α* ≥ 0.8 as good, and *α* ≥ 0.9 as excellent [17]. Since there is no ‘gold standard’ to measure IADL in brain tumour patients, the criterion validity could not be assessed. Instead, construct validation was examined by means of known-groups validity. An a priori hypothesis was constructed stating that patients classified as cognitively impaired would have higher scores (indicating more problems) on the IADL scales/items than patients who were classified as cognitively unimpaired. To do so, scale scores were calculated based on linear transformation as described in the EORTC Scoring Manual [18], and mean differences in the groups were compared using Mann–Whitney *U* tests. Congruency between patients and proxies was assessed per item (mean difference patient–proxy rating) and per dyad (inter-rater reliability) with sub-analyses including patients’ cognitive impairment classification (for more details, see Supplemental File 1). IBM SPSS version 26.0 was used to carry out all statistical analyses [19], and a *p* value < 0.05 was considered statistically significant.

## Results

Sociodemographic and clinical characteristics of patients and their proxies included in phases I and III are described in Table 2. In phase I, a total of 44 patients, 43 proxies, and 26 HCPs were included and in phase III, 85 dyads. Patients with and without cognitive impairments were fairly equal distributed among the tumour types (Table 3). Four patients could not complete the neuropsychological testing due to health issues.

**Table 2:** Sociodemographic and clinical characteristics of patients in both phase I and III, as well as the characteristics of their proxies

		Phase I			Phase III		
		Primary brain tumours	Metastatic brain tumours	All	Primary brain tumours	Metastatic brain tumours	All
Dyads (participants)	*N*	23 (45)	21 (42)	44 (87)	45 (90)	40 (80)	85 (170)
Region of residence	*N* (%)						
*Northern European region*		8 (35%)	8 (38%)	16 (36%)	19 (42%)	14 (35%)	33 (39%)
*Southern European region*		7 (30%)	9 (43%)	16 (36%)	5 (11%)	5 (13%)	10 (12%)
* English-speaking region*		8 (35%)	4 (19%)	12 (27%)	9 (20%)	9 (23%)	18 (22%)
*Non-European region*					12 (27%)	12 (30%)	24 (28%)
Sex (male)	*N* (%)						
*Patient*		14 (61%)	8 (38%)	22 (50%)	20 (44%)	12 (30%)	32 (38%)
*Proxy*		10 (45%)	12 (57%)	22 (51%)	19 (42%)	18 (45%)	37 (44%)
Age (yrs)	M (SD)						
*Patient*		55.39 (12.7)	60.75 (12.1)	57.95 (12.6)	54.68 (12.3)	61.48 (11.4)	57.88 (12.3)
*Proxy*		50.26 (14.7)	58.20 (13.2)	54.04 (14.4)	56.62 (10.5)	59.90 (12.9)	58.16 (11.7)
Level of education [1–8]* (Low [1–4])	*N* (%)						
*Patient*		16 (70%)	12 (57%)	28 (64%)	24 (53%)	25 (63%)	49 (58%)
*Proxy*		16 (73%)	14 (67%)	30 (70%)	23 (51%)	23 (58%)	45 (53%)
**Patients**							
Dominant hand (Right)	*N* (%)				37 (82%)	35 (88%)	72 (85%)
KPS	Median (range)				90 (60–100)	90 (40–100)	90 (40–100)
Histology	*N* (%)						
*LGG*		1 (4%)		1 (4%)	19 (22%)		19 (22%)
*HGG*		22 (96%)		22 (96%)	26 (31%)		26 (31%)
*BrM 1–3*			17 (81%)	17 (81%)		23 (27%)	23 (27%)
*BrM > 3*			4 (19%)	4 (19%)		17 (20%)	17 (20%)
Current active *N* (%) treatment					11 (24%)	18 (45%)	29 (34%)
*Chemotherapy*					8 (18%)	4 (10%)	12 (14%)
*Radiotherapy*					3 (7%)	9 (23%)	12 (14%)
-Stereotactic						2 (5%)	2 (5%)
-Whole brain						4 (10%)	4 (10%)
-Unspecified						3 (8%)	3 (8%)
*Other*					0 (0%)	5 (13%)	5 (6%)
Previous treatment *N* (%)							
*Resection*					37 (82%)	12 (30%)	49 (58%)
*Re-resection*					7 (16%)	2 (5%)	9 (11%)
* Chemotherapy*					26 (58%)	4 (10%)	30 (35%)
* Radiotherapy*					32 (71%)	29 (73%)	61 (72%)
-Stereotactic						20 (50%)	20 (50%)
-Whole brain						7 (18%)	7 (18%)
-Unspecified						1 (3%)	1 (3%)
*Other*					4 (9%)	1 (3%)	5 (6%)
Brain tumour hemisphere	*N* (%)						
*Left hemisphere*					15 (33%)	13 (33%)	28 (33%)
*Right hemisphere*					27 (60%)	10 (25%)	37 (44%)
*Midline*					2 (4%)	0 (0%)	2 (2%)
*Diffuse*					1 (2%)	17 (43%)	18 (21%)
Brain tumour location	*N* (%)						
*Frontal*					16 (36%)	10 (25%)	26 (31%)
*Temporal*					11 (25%)	1 (3%)	12 (14%)
*Occipital*					3 (7%)	3 (8%)	6 (7%)
*Parietal*					7 (16%)	1 (3%)	8 (9%)
* Intraventricular*					1 (2%)	1 (3%)	2 (2%)
*Multiple*					6 (14%)	24 (60%)	30 (35%)
Molecular biomarkers 1p/19q-codeletion	*N* (%)						
*1p/19q-codeletion*					6 (13%)		6 (13%)
*1p/19q non-codeletion*					8 (18%)		8 (18%)
*Unknown*					31 (69%)		31 (69%)
*IDH-mutation*							
*IDH-mutant*					8 (18%)		8 (18%)
*IDH-wildtype*					12 (27%)		12 (27%)
*Unknown*					25 (56%)		25 (56%)
Relation (Partner)	*N* (%)	17 (77%)	16 (76%)	33 (77%)	33 (73%)	28 (70%)	61 (72%)
Contact intensity	*N* (%)						
* Living together*		18 (82%)	17 (81%)	35 (81%)	38 (84%)	28 (70%)	66 (78%)
*Few times a week*		2 (9%)	1 (5%)	3 (7%)	6 (13%)	9 (23%)	15 (18%)
*Weekly*		2 (9%)	3 (14%)	5 (12%)	1 (2%)	3 (8%)	4 (5%)
Duration relationship (yrs)	M (SD)	28.55 (13.7)	32.58 (15.7)	30.46 (14.6)	31.38 (12.8)	34.83 (15.1)	33.00 (13.9)

*Level of education according to international standard classification of education ranging from 0 (low) to 8 (high) [20]

*yrs* years, *KPS* Karnofsky Performance Score, *LGG* low-grade glioma, *HGG* high-grade glioma, *BrM* brain metastases, *N* number, *M* mean, *SD* standard deviation

**Table 3:** Percentage of patients with cognitive impairments (defined as *z*-score more than 2 SD below the control group on at least two domains), separately per tumour type

Tumour type	Missing	Cognitively impaired	Not cognitively impaired
Primary BT		19 (42%)	24 (53%)
LGG (*n* = 19)	1 (5%)	8 (42%)	10 (53%)
HGG (*n* = 26)	1 (4%)	11 (42%)	14 (54%)
Brain metastases		18 (45%)	20 (50%)
BrM 1–3 (*n* = 23)	2 (9%)	11 (48%)	10 (43%)
BrM > 3 (*n* = 17)		7 (41%)	10 (59%)

*BT* brain tumour, *LGG* low-grade glioma, *HGG* high-grade glioma, *BrM* brain metastases

### Phase I & II

The review of the pilot study’s [9] activities (*n* = 32) reconfirmed that all items were considered IADL, affected in brain tumour patients and clearly defined. The literature search of 342 unique records resulted in 103 relevant records which described 54 unique questionnaires comprising a total of 1376 items. Out of these 1376 items, 310 were related to IADL. Furthermore, 23 IADL were extracted from qualitative studies. The review of the items in the EORTC Item Library identified 526 unique items of which 12 reflected IADL. The 345 IADL were clustered and merged based on content, and IADL similar to the pilot study items were excluded. On top of the 32 items from the pilot study (source 1), an additional 30 IADL were identified with sources 2 and 3, resulting in 62 activities. The semi-structured interviews generated two new activities (i.e. ‘being independent’ and ‘doing calculations’) and excluded two activities based on HCPs’ assessment of relevance (i.e. ‘arts and crafts’ and ‘following an instruction manual’). The remaining 62 activities were subsequently cognitively debriefed, resulting in the exclusion of two redundant activities (i.e. ‘putting ideas into words’ and ‘engaging socially with other people’) and one unclear activity (i.e. ‘getting started with a task without prompting’) (Fig. 1) (for more details, see Supplemental File 2). In phase II, the remaining 59 activities were formulated as items.

### Phase III

#### Missing activities

Nine activities suggested by participants could be considered IADL and were not covered by the 59-item list, however, none were mentioned by ≥ 2 participants, indicating that the item list had sufficient coverage.

#### Item selection

Following the item selection procedure, a total of 10 items were excluded in the first round, six items in the second round, and an additional 11 items in the third round (Fig. 1; for more details, see Supplemental File 3; Fig. 1). This item selection procedure resulted in a final list of 32 items.

##### Proxy ratings (sensitivity analysis)

Although item selection was based solely on patient data, the item selection procedure was repeated with proxy data. The 59 items from phase II were reduced to 33 items, 25 of which were the same as those selected based on patient data (data not shown).

#### Preliminary psychometric properties

##### Content validity

Most of the 32 items were deemed relevant and important; on average, items were rated as ‘quite a bit’ or ‘very much’ relevant by 57.2%, and important by 68.2% of patients. Following the acceptability criteria as described in Table 1, *N* = 24 items needed reviewing (for more details, see Supplementary file 3; Table 1). For twelve items, ≥ 2 dyads raised the same concern and were accordingly rephrased.

##### Structural validity

**Fig. 2:**
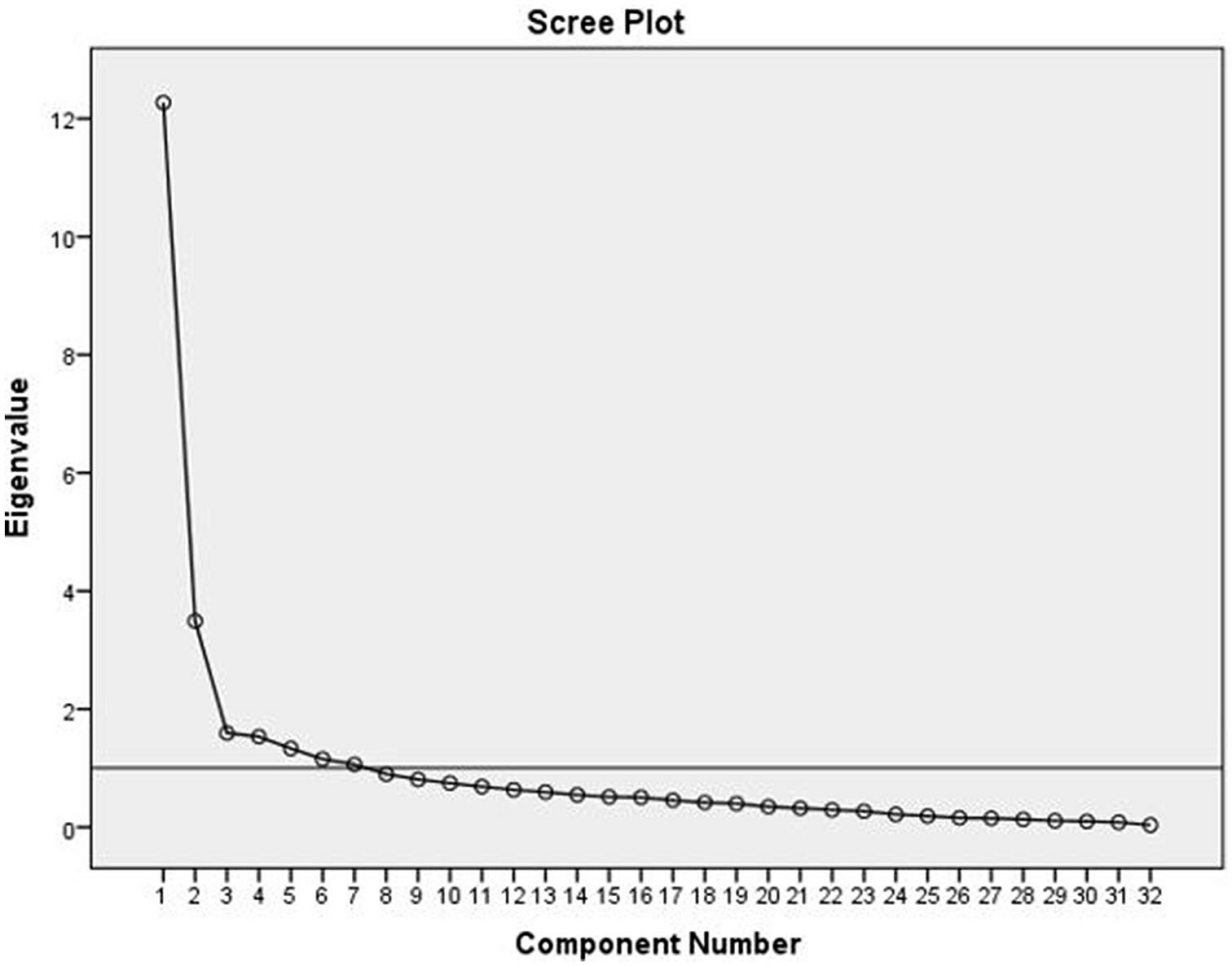
Scree plot

An EFA was conducted on the 32 IADL items. The Kaiser–Meyer–Olkin measure verified the adequacy of the pooled data: KMO = 0.816 and Bartlett’s test of sphericity was significant, *χ*^2^(496) = 1858.5, *p* < 0.001. Seven factors had an eigenvalue over Kaiser’s criterion of 1, which cumulatively explained 70.1% of the variance. The slope of the scree plot (Fig. 2), however, flattened after three factors. As this is an EFA, we decided to maintain the seven factors for further analyses. This resulted in five multi-item factors and two single-item factors (Table 4).

**Table 4:** IADL-BN32 preliminary scale structure: factor loadings of each item within each single- or multi-item scale

Scales	Items	Factor loadings
Scale 1	Have you had difficulty preparing a meal?	0.81
	Have you had difficulty doing maintenance chores in or around the house?	0.79
	Have you had difficulty performing household tasks (e.g. cleaning, doing laundry)?	0.78
	Have you had difficulty with grocery shopping?	0.78
	Have you had difficulty helping others, such as doing small chores or favours for a neighbour, friend or parent?	0.76
	Have you had difficulty performing your daily activities without help of others?	0.76
	Have you had difficulty taking care of family members (including children)?	0.72
	Have you had difficulty participating in traffic (e.g. crossing roads, or driving, cycling, or walking)?	0.68
	Have you had difficulty using household appliances?	0.62
	Have you had difficulty with your daily responsibilities (i.e. completing daily tasks such as cooking, cleaning, or shopping)?	0.57
	Have you had difficulty carrying out your hobbies?	0.48
Scale 2	Have you had difficulty expressing yourself (verbally or non-verbally)?	0.79
	Have you had difficulty switching between different activities that require concentration, such as watching television and participating in a conversation?	0.75
	Have you had difficulty finishing tasks that require concentration?	0.74
	Have you had difficulty recalling new information?	0.71
	Have you had difficulty managing your tasks (e.g. doing tasks in the right order)?	0.66
	Have you had difficulty following a one-on-one conversation?	0.57
	Have you had difficulty having a one-on-one conversation in noisy surroundings?	0.57
	Have you had difficulty finding important personal items (e.g. mobile phone or wallet) around the house?	0.45
Scale 3	Have you had difficulty using a computer/laptop/tablet?	0.73
	Have you had difficulty learning new things (e.g. to use a new appliance)?	0.70
	Have you had difficulty using a mobile telephone?	0.67
	Have you had difficulty writing (e.g. appropriate grammar)?	0.64
	Have you had difficulty reading?	0.55
Scale 4	Have you had difficulty making appointments?	0.72
	Have you had difficulty making decisions?	0.66
	Have you had difficulty doing your personal finances?	0.60
Scale 5	Have you had difficulty participating in social activities?	0.71
	Have you had difficulty participating in a group conversation?	0.67
	Have you had difficulty organizing a social activity (e.g. a dinner)?	0.58
Scale 6	Have you had difficulty managing your own medication?	0.73
Scale 7	Have you had difficulty doing your job (paid or voluntary)?	0.86

##### Internal consistency

The five multi-item factors, or preliminary scales, were evaluated for their internal consistency, which was excellent to acceptable, with Cronbach’s *α* of 0.94, 0.88, 0.83, 0.76, and 0.73, respectively.

##### Known-group validity

The known-group comparisons showed significant differences between cognitively impaired and unimpaired patients for scales 1 (ranked mean (RM) = 48.8 vs. RM = 34.4, *p* < 0.01), 3 (RM = 49.6 vs. RM = 33.1, *p* < 0.01), and 4 (RM = 46.9 vs. RM = 35.0, *p* = 0.02) scale, with worse performance in cognitively impaired patients. No significant differences between cognitively impaired and unimpaired patients were observed for scales 2 (RM = 43.8 vs. RM = 37.8, *p* = 0.24) and 5 (RM = 42.5 vs. RM = 35.6, *p* = 0.17), or the two single items 14 (RM = 18.6 vs. RM = 17.6, *p* = 0.78) and 45 (RM = 40.2 vs. RM = 35.2, *p* = 0.22).

##### Congruency

Proxies reported on average more problems more often than the patients (proxies: 26/32 vs. patients: 4/32), with an average mean difference of *M* = − 0.09 (SD = 0.11) [range *M* = − 0.27 to 0.26]. Patients with cognitive impairments had proxies reporting, on average, *M* = − 0.28 (SD = 0.20) more problems than the patients, while patient without cognitive impairments reported on average slightly more problems *M* = 0.03 (SD = 0.10) (for more details, see Supplemental File 3; Table 2). Furthermore, average exact agreement between patients and proxies was on average 56.7% [0–100%], with inter-rater agreement between *κ* = − 0.16 and *κ* = 1.00 (for more details, see Supplemental File 3; Table 3) (N/A excluded). For patients with and without cognitive impairments, this was 47.9% [*κ* = − 0.16 to 1.00] and 64.2% [*κ* = − 0.16 to 1.00], respectively.

## Discussion

The aim of this study is to develop a reliable and valid IADL measure for brain tumour patients. Following the procedures of phases I–III of the EORTC module development guidelines, ensuring content and cross-cultural validity, resulted in the construction of the EORTC IADL-BN32 questionnaire. Preliminary psychometric property analyses showed that the questionnaire has a multidimensional scale structure with acceptable to excellent internal consistency. Moreover, the current scale structure showed known-groups validity regarding cognitive status for three out of five scales in this relatively small sample. Congruency between patients and proxies showed quite some variation between dyads. Agreement between cognitively impaired patients and their proxies was on average lower, with their proxies rating IADL issues as slightly more severe, compared to cognitive unimpaired patients. Therefore, the patient and proxy versions of the EORTC IADL-BN32 will be further assessed in phase IV as it is still unclear if the proxy-based questionnaire is more accurate and preferable in situations where patients are cognitively impaired or in poor health.

Limitations of this study were the relatively small sample and the skewed number of participants per geographical region due to participant recruitment issues at some sites. Results should therefore be interpreted as preliminary, and further validation in a larger sample in phase IV is warranted. A predetermined 3-round stepwise item selection procedure was implemented to compensate for this imbalance and ensure cross-cultural validity. Furthermore, many patients and proxies reported ‘not applicable’ on one or more items resulting in a large proportion of missing data, hampering the EFA. This was corrected by means of multiple imputation. To facilitate the CFA in the phase IV validation, as well as the calculation of the scale/item scores of the final questionnaire, this response option will be omitted in further versions. CFAs in phase IV will confirm if the preliminary scale structure is more accurate than, for example, a single factor model. Finally, item selection in phase III was based on patient data only, while cognitive impairment may result in poorer self-awareness of IADL issues [21]. In our sample, however, the sensitivity analyses with proxy data showed that 25/32 of the same items would have been selected.

In conclusion, the preliminary EORTC IADL-BN32 questionnaire has reasonable preliminary psychometric properties, however, further validation in a larger international sample is warranted. The phase IV validation is currently ongoing in ten countries in different global regions. Additional European countries (i.e. Germany, Norway, Portugal, and Croatia) as well as an additional non-European country (i.e. Jordan) are participating in the phase IV validation, further enhancing the generalizability of our results. The focus of phase IV will be on evaluating the scale structure, responsiveness over time, and acceptability of the questionnaire. If the EORTC IADL-BN32 is valid and reliable, it may be a valuable tool in brain tumour trials and clinical practice to monitor levels of functioning in daily life and may be helpful in evaluating the day-to-day impact of changes in cognitive function.

## Supplementary Information

Below is the link to the electronic supplementary material.

Supplementary file1 (DOCX 218 KB)

## References

[1] Lawton MP, Brody EM (1969). Assessment of older people: Self-maintaining and instrumental activities of daily living. Gerontologist.

[2] Passler JS, Kennedy RE, Clay OJ (2020). The relationship of longitudinal cognitive change to self-reported IADL in a general population. Aging, Neuropsychology, and Cognition.

[3] Koster N, Knol DL, Uitdehaag BM, Scheltens P, Sikkes SA (2015). The sensitivity to change over time of the Amsterdam IADL Questionnaire((c)). Alzheimers Dement.

[4] Bosma I, Vos MJ, Heimans JJ (2007). The course of neurocognitive functioning in high-grade glioma patients1. Neuro-Oncology.

[5] Oort Q, Taphoorn MJB, Sikkes SAM, Uitdehaag BMJ, Reijneveld JC, Dirven L (2019). Evaluation of the content coverage of questionnaires containing basic and instrumental activities of daily living (ADL) used in adult patients with brain tumors. Journal of Neuro-oncology.

[6] Sikkes SA, de Lange-de Klerk ES, Pijnenburg YA (2012). A new informant-based questionnaire for instrumental activities of daily living in dementia. Alzheimers Dement.

[7] Sikkes SA, Knol DL, Pijnenburg YA, de Lange-de Klerk ES, Uitdehaag BM, Scheltens P (2013). Validation of the Amsterdam IADL Questionnaire(c), a new tool to measure instrumental activities of daily living in dementia. Neuroepidemiology.

[8] Sikkes SA, Pijnenburg YA, Knol DL, de Lange-de Klerk ES, Scheltens P, Uitdehaag BM (2013). Assessment of instrumental activities of daily living in dementia: Diagnostic value of the Amsterdam Instrumental Activities of Daily Living Questionnaire. Journal of Geriatric Psychiatry and Neurology.

[9] Oort Q, Dirven L, Meijer W (2017). Development of a questionnaire measuring instrumental activities of daily living (IADL) in patients with brain tumors: A pilot study. Journal of Neuro-Oncology.

[10] Gehrke AK, Baisley MC, Sonck ALB, Wronski SL, Feuerstein M (2013). Neurocognitive deficits following primary brain tumor treatment: Systematic review of a decade of comparative studies. Journal of Neuro-Oncology.

[11] Hugo J, Ganguli M (2014). Dementia and cognitive impairment: Epidemiology, diagnosis, and treatment. Clinics in Geriatric Medicine.

[12] Fayers P, Machin D (2000). Quality of life. Assessment, analysis and interpretation.

[13] Howorth P, Saper J (2003). The dimensions of insight in people with dementia. Aging & Mental Health.

[14] Johnson, C., Aaronson, & N., Blazeby, J. M., et al. (2011). EORTC Quality of Life Group: Guidelines for developing questionnaire modules (4th ed.)*. *EORTC Quality of Life Group.

[15] EORTC QOL Item Library. https://qol.eortc.org/item-library/.

[16] Kulis, D., Bottomley, A., Velikova, G., Greimel, E., & Koller, M. (2017) EORTC Quality of Life Group Translation Procedure. 10.1080/14737167.2017.1384316 28974101

[17] Kline, P. (2000). *The handbook of psychological testing*. Psychology Press..

[18] EORTC QLQ-C30 Scoring Manual. *EORTC QOL Group*. (2001).

[19] IBM Corp. IBM SPSS Statistics for Windows, Version 24.0. (2016).

[20] Schneider, S.L. (2011). The International Standard Classification of Education 2011. in* Class and Stratification Analysis*, pp. 365–379.

[21] Ediebah DE, Reijneveld JC, Taphoorn MJ (2017). Impact of neurocognitive deficits on patient–proxy agreement regarding health-related quality of life in low-grade glioma patients. Quality of Life Research.

