# Supplementary material for: Development of an EORTC questionnaire measuring instrumental activities of daily living (IADL) in patients with brain tumours: phase I–III
Source: Qual Life Res. 2021 Jan 26;30(5):1491–502. doi: 10.1007/s11136-020-02738-5 (PMC8068708; doi:10.1007/s11136-020-02738-5)
Supplement: Supplementary file 1 — Supplementary file1 (DOCX 218 KB) [file 11136_2020_2738_MOESM1_ESM.docx]

**Supplemental Files**

**Supplemental File 1. Detailed methods phases I-III**

**Phase I**

The participant population in phase I consisted of both patients with a primary and metastatic brain tumour, their proxies and HCPs experienced in the field of neuro-oncology, from three different countries (The Netherlands, United Kingdom, Italy).

Step 1. Pilot study: Patients and their proxies, as well as HCPs, were recruited to review a list of 32 items resulting from the previously conducted study in glioma patients^9^. All participants were asked to answer two questions: ‘Is the activity likely to be affected in brain tumour patients?’ and ‘Is the item clearly defined and formulated?’ Moreover, if participants considered items to be unclear, they were requested to substantiate their answer. Furthermore, HCPs answered one additional question: ‘Is the activity considered as IADL using the proposed definition (=‘IADL are complex activities with little automated skills for which multiple cognitive processes are necessary’^6^)?’.

The predetermined decision rules regarding item exclusion were as follows;

• Question 1: Can the activity be considered as IADL?

An activity was excluded if ≥2/6 HCPs responded that the activity did not correspond with proposed definition of IADL.

• Question 2: Affected in brain tumour patients?

An activity was excluded if <3/6 HCPs, <3/12 patients or <3/12 did not recognize the activity as being affected in brain tumour patients.

• Question 3: Clearly defined and formulated?

The formulation of the activity was considered unclear if ≥2/6 HCPs, ≥2/12 patients or ≥2/12 proxies considered the item to be unclear.

Activities were retained if they were either considered (a) IADL, affected and clearly defined or (b) IADL and affected, but not clearly defined. In the latter case, the items were rephrased in accordance with the input provided by the participants.

Step 2. Literature review: A literature review of the electronic databases PubMed, Embase, Cochrane, PsycINFO and CINAHL was conducted up to April 2017, and the EORTC Item Library was searched for possible relevant items. The search string for the literature search consisted of a combination of three components, one related to brain tumours and one related to (I)ADL.

**Search string for Pubmed/Medline for the systematic literature in phase I:**

(("Glioma"[Mesh] OR glioma*[ot] OR glioma*[tiab] OR glio-ma*[tiab] OR gli-oma*[tiab] OR glyoma*[tiab] OR glyo-ma*[tiab] OR glyoma*[tiab] OR neuroglioma*[tiab] OR neuroglyoma*[tiab] OR ((neoplasm*[ti] OR neoplasm*[ti] OR tumor*[ti] OR tumour*[ti] OR cancer*[ti] OR malignan*[ti]) AND (glia*[ti] ORneuroglia*[ti])) OR glial neoplasm*[tiab] OR "glial neo-plasm"[tiab] OR "glial neo-plasms"[tiab] OR glial cell neoplasm*[tiab] OR "glial cell neo-plasm"[tiab] OR "glial cell neo-plasms"[tiab] OR glial brain neoplasm*[tiab] OR "glial brain neo-plasm"[tiab] OR "glial brain neoplasms"[tiab] OR "glial cns neoplasm"[tiab] OR "glial cns neo-plasm"[tiab] OR "glial cns neoplasms"[tiab] OR "glial cns neo-plasms"[tiab] OR glial tumor*[tiab] OR glial tumour*[tiab] OR glial cell tumor*[tiab] OR glial cell tumour*[tiab] OR glial brain tumor*[tiab] OR glial brain tumour*[tiab] OR "glial cns tumor"[tiab] OR "glial cns tumors"[tiab] OR "glial cns tumour"[tiab] OR "glial cns tumours"[tiab] OR glial cancer*[tiab] OR "glial cell cancer"[tiab] OR "glial cell cancers"[tiab] OR "glial brain cancer"[tiab] OR "glial brain cancers"[tiab] OR "glial cns cancer"[tiab] OR "glial cns cancers"[tiab] OR glial malign*[tiab] OR "glial cell malignancy"[tiab] OR "glial cell malignancies"[tiab] OR "glial brain malignancy"[tiab] OR "glial brain malignancies"[tiab] OR "glial cns malignancy"[tiab] OR "glial cns malignancies"[tiab] OR malignant glia*[tiab] OR malignant glial *[tiab] OR xanthoastrocytoma*[tiab] OR xantoastrocytoma*[tiab] OR astrocytoma*[tiab] OR astro-cytoma*[tiab] OR astroglioma*[tiab]

OR astro-glioma*[tiab] OR oligoastrocytoma*[tiab] OR oligoastro-cytoma*[tiab] OR glioblastom*[tiab] OR glio-blastom*[tiab] OR oligodendroglioma*[tiab] OR oligodendroglioma*[tiab] OR oligoden-droglioma*[tiab] OR oligodendroblastoma*[tiab] OR oligodendro-blastoma*[tiab] OR oligo-dendroblastoma*[tiab] OR oligoden-droblastoma*[tiab] OR oligodendro-blastoma*[tiab] OR ependymom*[tiab] OR ependimom*[tiab] OR subependymom*[tiab] OR subependimom*[tiab] OR gliosarcoma*[tiab] OR gliosarcoma*[tiab] OR brain malign*[tiab] OR malignant primary brain*[tiab] OR primary malignant brain*[tiab] OR malignant brain*[tiab]) OR ("Brain Neoplasms"[Mesh] OR tumour of brain*[tiab] OR tumor of brain*[tiab] OR "tumours of brain"[tiab] OR "tumors of brain"[tiab] OR "tumours of brainstem"[tiab] OR "tumors of brainstem"[tiab] OR tumour of the brain*[tiab] OR

tumor of the brain*[tiab] OR "tumours of the brain"[tiab] OR "tumors of the brain"[tiab] OR "tumours of the brainstem"[tiab] OR "tumors of the brainstem"[tiab] OR brain tumour*[tiab] OR brain tumor*[tiab] OR brainstem tumour*[tiab] OR brainstem tumor*[tiab] OR brain-stem tumour*[tiab] OR brain-stem tumor*[tiab] OR frontal tumour*[tiab] OR frontal tumor*[tiab] OR "tumour of cns"[tiab] OR tumor of cns*[tiab] OR "tumours of cns"[tiab] OR "tumors of cns"[tiab] OR tumour of the cns*[tiab] OR tumor of the cns*[tiab] OR "tumours of the cns"[tiab] OR "tumors of the cns"[tiab] OR cns tumour*[tiab] OR cns tumor*[tiab] OR "tumour of central nervous system"[tiab] OR tumor of central nervous system*[tiab] OR "tumours of central nervous system"[tiab] OR "tumors of central nervous system"[tiab] OR tumour of the central nervous system*[tiab] OR tumor of the central nervous system*[tiab] OR "tumours of the central nervous system"[tiab] OR "tumors of the central nervous system"[tiab] OR central nervous system tumour*[tiab] OR central nervous system tumor*[tiab] OR intracranial tumour*[tiab] OR intracranial tumor*[tiab] OR intra-cranial tumour*[tiab] OR intra-cranial

tumor*[tiab] OR cerebral tumour*[tiab] OR cerebral tumor*[tiab] OR intracerebral tumour*[tiab] OR intracerebral tumor*[tiab] OR neoplasm of brain*[tiab] OR "neoplasms of brain"[tiab] OR "neoplasms of brainstem"[tiab] OR neoplasm of the brain*[tiab] OR "neoplasms of the brain"[tiab] OR "neoplasms of the brainstem"[tiab] OR brain neoplasm*[tiab] OR brainstem neoplasm*[tiab] OR brain-stem neoplasm*[tiab] OR frontal neoplasm*[tiab] OR neoplasm of cns*[tiab] OR "neoplasms of cns"[tiab] OR neoplasm of the cns*[tiab] OR "neoplasms of the cns"[tiab] OR cns neoplasm*[tiab] OR "neoplasm of central nervous system"[tiab] OR "neoplasms of central nervous system"[tiab] OR neoplasm of the

central nervous system*[tiab] OR "neoplasms of the central nervous system"[tiab] OR central nervous system neoplasm*[tiab] OR intracranial neoplasm*[tiab] OR intra-cranial neoplasm*[tiab] OR cerebral neoplasm*[tiab] OR intracerebral neoplasm*[tiab] OR cancer of brain*[tiab] OR "cancers of brain"[tiab] OR "cancers of brainstem"[tiab] OR cancer of the brain*[tiab] OR "cancers of the brain"[tiab] OR "cancers of the brainstem"[tiab] OR brain cancer*[tiab] OR "brainstem cancer"[tiab] OR "brainstem cancers"[tiab] OR "brain-stem cancer"[tiab] OR "brain-stem cancers"[tiab] OR "frontal cancer"[tiab] OR "frontal cancers"[tiab] OR "cancer of cns"[tiab] OR "cancers of cns"[tiab] OR "cancer of the cns"[tiab] OR "cancers of the cns"[tiab] OR cns cancer*[tiab] OR "cancer of central nervous system"[tiab] OR "cancers of central nervous system"[tiab] OR cancer of the central nervous system*[tiab] OR "cancers of the central nervous system"[tiab] OR central nervous system cancer*[tiab] OR intracranial cancer*[tiab] OR "intra-cranial cancer"[tiab] OR "intra-cranial cancers"[tiab] OR cerebral cancer*[tiab] OR "intracerebral cancer"[tiab] OR "intracerebral cancers"[tiab] OR "carcinoma of brain"[tiab] OR "carcinomas of brain"[tiab] OR "carcinoma of brainstem"[tiab] OR "carcinomas of brainstem"[tiab] OR "carcinoma of the brain"[tiab] OR "carcinomas of the brain"[tiab] OR "carcinoma of the brainstem"[tiab] OR "carcinomas of the brainstem"[tiab] OR brain carcinoma*[tiab] OR "brainstem carcinoma"[tiab] OR "brainstem carcinomas"[tiab] OR "brain-stem carcinoma"[tiab] OR "brain-stem carcinomas"[tiab] OR "frontal carcinoma"[tiab] OR "frontal carcinomas"[tiab] OR "carcinoma of cns"[tiab] OR "carcinomas of cns"[tiab] OR "carcinoma of the cns"[tiab] OR "carcinomas of the cns"[tiab] OR "cns carcinoma"[tiab] OR "cns carcinomas"[tiab] OR "carcinoma of central nervous system"[tiab] OR "carcinomas of central nervous system"[tiab] OR "carcinoma of the central nervous system"[tiab] OR "carcinomas of the central nervous system"[tiab] OR "central nervous system carcinoma"[tiab] OR "central nervous system carcinomas"[tiab] OR "intracranial carcinoma"[tiab] OR "intracranial carcinomas"[tiab] OR "intra-cranial carcinoma"[tiab] OR "intra-cranial carcinomas"[tiab] OR cerebral carcinoma*[tiab] OR

"intracerebral carcinoma"[tiab] OR "intracerebral carcinomas"[tiab] OR "malignancy of brain"[tiab] OR "malignancies of brain"[tiab] OR "malignancy of brainstem"[tiab] OR "malignancies of brainstem"[tiab] OR "malignancy of the brain"[tiab] OR "malignancies of the brain"[tiab] OR "malignancy of the brainstem"[tiab] OR "malignancies of the brainstem"[tiab] OR brain malignan*[tiab] OR "brainstem malignancy"[tiab] OR "brainstem malignancies"[tiab] OR "brain-stem malignancy"[tiab] OR "brain-stem malignancies"[tiab] OR "frontal malignancy"[tiab] OR "frontal malignancies"[tiab] OR "frontal malignant"[tiab] OR "malignancy of cns"[tiab] OR "malignancies of cns"[tiab] OR "malignancy of the cns"[tiab] OR "malignancies of the cns"[tiab] OR cns malignan*[tiab] OR "malignancies of central nervous system"[tiab] OR "malignancy of central nervous system"[tiab] OR "malignancy of the central

nervous system"[tiab] OR "malignancies of the central nervous system"[tiab] OR central nervous system malignan*[tiab] OR intracranial malignan*[tiab] OR intra-cranial malignan*[tiab] OR cerebral malignan*[tiab] OR intracerebral malignan*[tiab])) **AND** Activities of daily living (MeSH), iadl, instrumental adl, instrumental activities of daily living, extended ADL, complex ADL, advanced ADL, functional ability, everyday functioning and activities of daily living.

Step 3. Semi-structured interviews: A new group of patients, proxies and HCPs underwent the semi-structured interviews to generate new activities not yet covered by the activities generated from the literature review and pilot study. In-depth semi-structured open question interviews were conducted using the ‘sampling to redundancy’ criterion (in which the participants were interviewed until no new activities emerged^21^). Only the activities that were in accordance with the proposed IADL definition and mentioned by two or more HCPs or patients and proxies were included. In addition, each activity identified in the literature review and pilot study was rated on both relevance and importance (4‐point Likert‐scale, ranging from ‘not at all’ to ‘very much’) by patients and proxies separately. HCPs also rated the activities on relevance and additionally provided a top 10 of the most important activities. To reduce the number of items for phase III, only relevant and important items were retained. Items with an average score of <2.0 on both relevance and importance by either the patients or proxies, or with ≥6 HCPs rating them as not relevant, were excluded, except if they were in ≥2 HCPs 10 top most important.

Step 4. Cognitive debriefing: Again a new group of patients, proxies and HCPs cognitively debriefed the relevant and important IADL from the literature and the pilot study, and the newly generated activities from the semi-structured interviews. They were requested to complete the new set of activities while thinking out loud. This technique was used to test whether all activities were interpreted as intended. Predetermined decision rules stated that if ≥2 participants indicated similar mentions of ambiguity or repetitiveness then the activity would be rephrased, merged or omitted. All suggestions mentioned by the participants were taken into consideration and if valid, adjusted accordingly.

**Phase II**

In phase II, the activities from phase I were converted into items and constructed in two versions of the draft questionnaire: a patient‐based version and a proxy‐based version. The formulation of the items was similar to the items in other EORTC questionnaires, and the response format was also the same (i.e. a 4‐point Likert scale ranging from ‘not at all’ to ‘very much’). In addition, the response option ‘not applicable’ was added to each question if not relevant for that patient in that timeframe. Moreover, questions refer to the patient’s experience during the past month (and not the past week), because many problems are unlikely to be captured within a one-week timeframe. Translations of all items into the languages of the countries participating in phase III (English, Dutch, Italian, German and Japanese) were carried out by the EORTC Translation Unit.

**Phase III**

Phase III consisted of two components, (1) completion of the semi-structured interview, and (2) neurocognitive testing. During the interview, participants had to complete four parts. In part 1 of the interview, brain tumour patients (patient‐based version) and their proxies (proxy‐based version) completed the 59-item questionnaire that resulted from phase I and II. In part 2, participants were asked to rate each item on both relevance and importance on a 4‐point Likert‐scale. In addition, participants were asked to rate if each question was acceptable (i.e. not too difficult, confusing, annoying or upsetting). In part 3 of the interview, patients and proxies were asked to identify the 10 activities that were most important to them and should definitely be included in the final questionnaire. Lastly, in part 4 of the interview, patients and proxies were asked to indicate if the questionnaire was complete or if certain activities were missing, by means of one general open question. Data from the semi-structured interviews will be analysed both qualitatively and qualitatively.

The second component of phase III was neuropsychological testing of the patients. The neuropsychological test battery was comprised of three objective tests (the Hopkins Verbal Learning Test–Revised (HVLT-R)^22^, Trail Making Test (TMT) A+B^23^, Controlled Oral Word Association Test (COWAT) adjusted for per language)^24^ with six outcome measures, and the MOS Cognitive Functioning Scale–Revised (MOS COG–R)^25^ questionnaire. To calculate the patients’ level of cognitive performance on the TMT A+B and COWAT, norm scores from Mitrushina, Boone, Razani, & D'Elia (2005) were used^26^. Norm scores for the HVLT-R were derived from the Hopkins Verbal Learning Test–Revised Professional Manual^22^. The MOS COG–R norm scores were derived from the User’s Manual for the Medical Outcomes Study (MOS) Core Measures of Health-Related Quality of Life^25^. Patients with z-score of more than 2.0 standard deviation (SD) below the control group on at least two neuropsychological outcome measures were classified as cognitively impaired.

Statistical analysis

The ratings of level of difficulties with the IADL, the relevance and importance (number and percentage of responses on 4-point Likert scale, range) and the 10 most important items for each item for each geographic region were assessed using descriptive statistics.

*Item selection*

Item selection rules were selected as proposed in the EORTC Quality of Life Group (QLG) module development guidelines. A stepwise procedure of item selection was conducted (see Supplemental File 1; Figure 1), to ensure cross-cultural validity (due to the imbalance in the number of patients in the different geographical regions) and content validity were maintained. Three selection rounds were constructed.

*Round 1*

Firstly, items were selected per geographical region:

1) the top 10 items out of the ‘10 most important items’ (part 3 of the interview) as rated by patients,

2) additional items with ≥60% of the patients rating both importance and relevance with scores 3 or 4,

3) additional items with mean score >2 on the items (N/A responses excluded).

*Round 2*

All items meeting the selection criteria in round 1 were included the second round. Unless the item was one of the 10 most important items rated by patients, items were excluded if:

4) <60% of the patients rated both importance and relevance with scores 3 or 4 in more than one geographical region,

5) ≥75% of the patients rated the item as ‘Not applicable’ in more than one geographical region.

*Round 3*

Unless the item was one of the 10 most important items rated by patients in two or more geographical regions, items were excluded if:

7) prevalence ratio <30% (sum of scores 2, 3 or 4/number of participants)

8) an item had floor- or ceiling effects (responses in categories 1&2 or 3&4 were >20%)

9) range of the item score ≤2 points

10) item completion rate ≤95%

*Content validity*

The percentage of patients rating the items as ‘quite a bit’ or ‘very much’ relevant and ‘quite a bit’ or ‘very much’ important was assessed for each item after item selection. Furthermore, if >5% of participants (85 patients and 85 proxies = 170 = 8.5 participants as cut-off) or >5 participants from a single geographical region rated an item as ‘Difficult / Confusing / Annoying / Upsetting’, this item was more closely reviewed. If ≥2 dyads raised the same concern, then this was addressed by rephrasing the item or, if this was not possible, excluding the item.


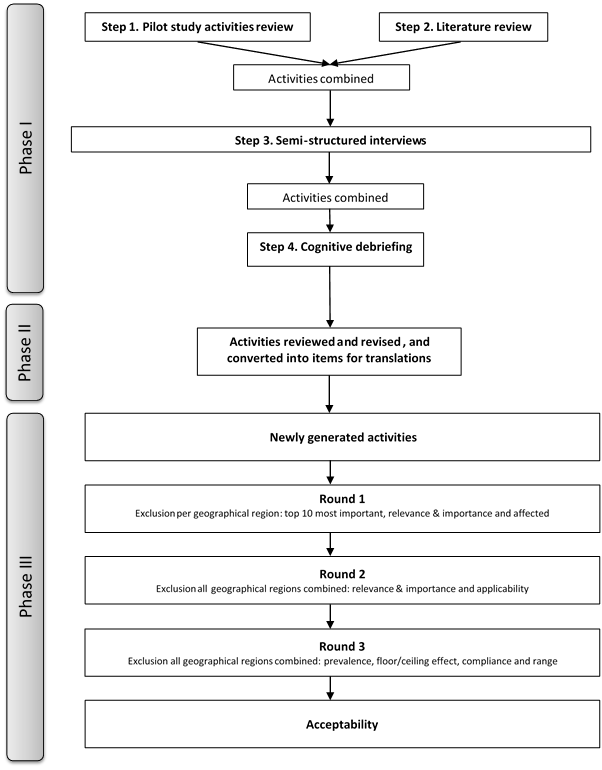


**Supplemental File 1; Figure 1. Flowchart of the module development process**

*Structural validity*

All ‘not applicable’ responses were assessed and recoded as missing data. Since the listwise cases exclusion of missing data in the standard EFA which would severely limit the number of patients included in the analysis, multiple imputation (MI) was used. Ten imputations with Markov Chain Monte Carlo (MCMC) were performed, running 100 iterations and using predictive mean matching (PMM) for imputation. The predictors included in the MI were age, sex, level of education (0-8), geographical region, tumour type (low grade glioma, high grade glioma, 1-3 brain metastases and >3 brain metastases), cognitive impairment (yes/no) and Karnofsky Performance Status score.

After item selection, structural validity was assessed by performing an EFA (principal component analysis) with orthogonal rotation (varimax). The Kaiser-Meyer-Olkin test, a measure of the proportion of variance among variables that might be common variance, and the Bartlett’s test of sphericity, measuring if the correlation structure is adequate for factor analyses, were performed. Items with a factor loading of ≥0.3 on at least one factor were included.

*Internal consistency*

The internal consistency of the preliminary multi-item scales resulting from the EFA was determined with Cronbach’s alpha, and alphas between 0.8> α ≥0.7 were classified as acceptable, 0.9> α ≥0.8 as good and α ≥0.9 as excellent.

*Known-groups validity*

Since there is no ‘gold standard’ to measure IADL in patients with brain tumours, the criterion validity could not be assessed and therefore construct validation examined performed by means of known-groups. This was determined by means of known-group comparisons (patients with cognitive deficits versus patients without cognitive deficits, as measured with neuropsychological testing). For the known-group comparisons, scores for the scales resulting from the EFA were calculated, based on the calculation for linear transformation as described in the EORTC QLQ-C30 Scoring Manual^18^.

*Congruency*

Patient ratings were subtracted from proxy ratings to get the mean difference between the two participant groups to evaluate if items are rated, on average, higher by patients or proxies. Positive mean difference indicating higher ratings on IADL items from patients and negative mean difference indicating higher ratings by their proxies. Furthermore, raw agreement and interrater agreement (Cohen’s kappa) between the dyads were assessed (N/A excluded).

Qualitative analysis

The exact rephrasing of the items resulting from the EFA was based on the qualitative comments that participants provided during the cognitive debriefing (part 2 of the interview). Moreover, qualitative data was also used for the potential inclusion of new issues (part 4 of the interview): if >2 dyads mentioned the same new issue that could be considered IADL, the item was eligible for inclusion.

**Supplemental File 2.**

**Phase I&II; Detailed results**

Step 1. Pilot study:

- Question 1: Can the activity be considered as IADL?

Six HCPs evaluated if the activities in the pilot study item list could be considered as IADL. Based on the predetermined cut-off criteria, all activities could be considered as IADL and were therefore retained.

- Question 2: Are the activities affected in brain tumour patients?

Six HCPs, six primary brain tumour patients, six brain metastases patients and their twelve proxies evaluated if activities in the provisional item list were affected in brain tumour patients. All activities, except one, were considered as likely to be affected in brain tumour patients by HCPs, based on the predetermined cut-off criteria. Two HCPs indicated that they had some doubts about the activity ‘usage of a navigation system’; one HCP was not clear whether this activity is affected in brain tumour patients and the other HCP indicated that the item is too specific and not many people own a navigation system. The patients and proxies recognized all (32/32, 100%) and almost all activities (31/32; 97%), respectively, as likely to be affected. All activities were considered likely to be affected in brain tumour patients by either patients, proxies or HCPs, and were therefore retained.

- Question 3: Are the activities clearly defined and formulated?

All 30 participants evaluated if the items in the provisional item list were clearly defined and formulated. Participants were asked to substantiate their answer if they deemed the item unclear. Based on the predetermined cut-off criteria, HCPs regarded 17/32 (53%) activities of the items as clear, whereas, the patient- and proxy group both regarded 29/32 (91%) of the items as clear. If ≥2 participants (HCPs, patients or proxies) had similar comments, the item would be altered accordingly. Several items (n=15) were rephrased based on the provided comments.

The activities in the provisional item list could all be considered either (a) IADL, affected and clearly defined or (b) IADL and affected, but not clearly defined.

Step 2. Literature review: The literature review identified 456 records through database searching. After removal of duplicates, 342 records were remained. Subsequently, records were excluded if: the study population did not include patients with a glioma or brain metastases (n=93), if patients were <18 years or with childhood acquired brain tumours (n=68), was non peer-reviewed (e.g. editorials) (n=11), the article was not written in English (n=9), animal/cell line studies (n=2) and own pilot study (n=1) based on title and abstract only. Full text records were excluded if: the study did not report on (identifiable) (I)ADL/QOL/Functional measures or only included measures of performance scale (n=29) or BADL (e.g. Karnofsky Performance Status; Barthel Index) (n=21) or when no full text was available (n=5). From the remaining 103 records, 54 unique (I)ADL/QOL/Functional questionnaires were identified and five qualitative interviews with (I)ADL items (Supplemental File 2; Figure 1).

In total, 1376 items were extracted from the 54 questionnaires, of which 310 contained instrumental activities of daily living, and 23 instrumental activities of daily living from qualitative studies. The EORTC QLG item library contained 33 modules besides the EORTC QLQ-C30 and QLQ-BN20 (already included through literature review) with 784 items of which 526 items with unique content. Of these 526 items, 12 items reflected instrumental activities of daily living. The total of 345 items (310 + 23+ 12) pertaining IADL were extracted. Items with the same or similar activities were merged and items with IADL already covered by the provisional item list that resulted from the pilot study were excluded, resulting in 30 new unique activities in addition to the provisional item list.

Full-text articles excluded (*n*=**55**)

No (I)ADL/QOL measure n=29

Only BADL measure n=21

Full text unavailable n=5

Records excluded (*n* = **184**)

No brain tumor patients n=93

Childhood brain tumor n=68

Non peer-reviewed articles n=11

Not in English n=9

Animal/cell line studies n=2

Own study n=1

Records identified through database searching
(*n* = **456** PubMed, Embase, Cochrane, PsychINFO, CINAHL)

)

Final list of studies included
(*n* = **103**)

Full-text articles assessed for eligibility
(*n* =**158**)

Records after duplicates removed

(*n* = **342**)

(*n* = **158**)

plicates removed
(*n* = **342**)

Screening

**Records identified**

Included

Eligibility

**Supplemental File 2; Figure 1. Schematic breakdown of literature search results**

Step 3. Semi-structured interviews: During the interviews with 28 patients (n=15 primary, n=13 metastatic brain tumour patients), 27 proxies and 18 HCPs, several new activities were mentioned. However, most were either not in accordance with the proposed IADL definition and/or mentioned by two or more HCPs, patients or proxies, and therefore not eligible for inclusion. Only two new activities were derived from the interviews and included in the item list; problems with ‘being independent’ and ‘doing calculations’.

Step 1 and 2 (literature review and review provisional item list) resulted in 62 eligible activities that were assessed for relevance and importance. Based on the predefined cut-off criteria, two activities were rated as irrelevant by 13/18 (72%) HCPs; difficulties with ‘following an instruction manual’ and ‘arts & crafts’. The item on ‘arts and crafts’ was also not deemed important or relevant by proxies. Therefore, these two activities were excluded, resulting in a list of 62 activities.

Step 4. Cognitive debriefing: Four patients (n=2 primary and n=2 metastatic brain tumour), four proxies and two HCPs underwent the cognitive debriefing. The cognitive debriefing resulted in several minor alterations of items, reformulations or items were clarified with examples (total n=24 items). Furthermore, three activities were omitted; 1. ‘Putting ideas into words’ was omitted because it overlapped with ‘Expressing yourself (verbally or non-verbally)’ (which was said to be preferable), 2. ‘Engaging socially with other people?’ was overlapping with its previous item ‘Maintaining personal relationships (e.g. visiting/phoning or writing with friends or relatives)?’ and changed into ‘Keeping in touch with family/friends?’, and 3. ‘Getting started with a task without prompting?’ was considered too broad and complicated to be rephrased and therefore excluded. In conclusion, a total of 59 items remained (62 – 3 = 59).

**Supplemental File 3.**


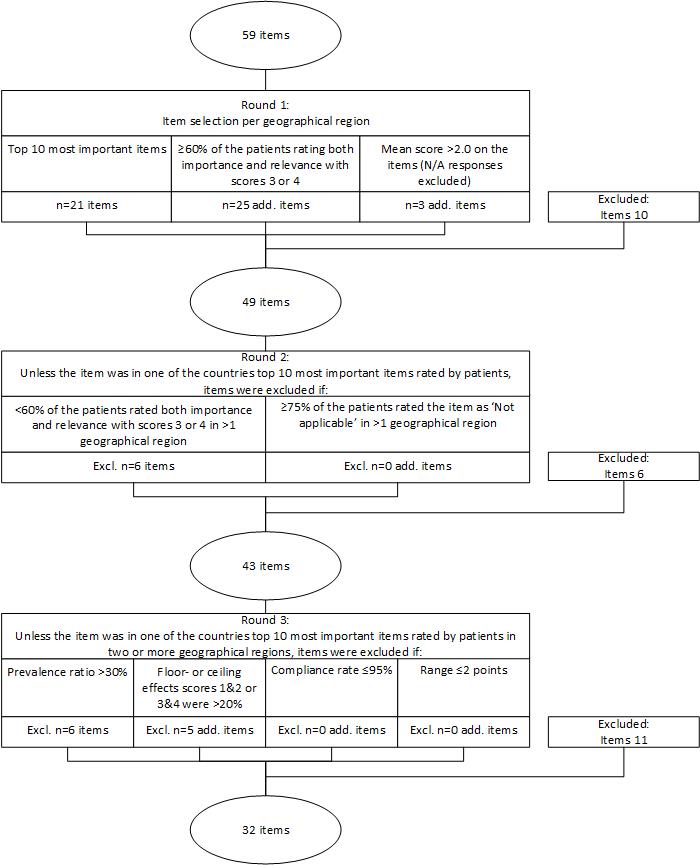
**Phase III; Detailed results**

**Supplemental File 3; Fig 1. Detailed flowchart of phase III patients’ item selection procedure.** Abbreviations: add. = additional, Excl. = excluded

| Item |  | Confusing | Difficult | Annoying | Upsetting | Total |
| --- | --- | --- | --- | --- | --- | --- |
|  |  | N (%) | N (%) | N (%) | N (%) | N (%) |
| Item 1˜ | Northern European region | 3 (1.8%) | - | - | - | 3 (1.8%) |
|  | Southern European region | - | - | - | - | - |
|  | English speaking region | 3 (1.8%) | 3 (1.8%) | 8 (4.7%) | 2 (1.2%) | **16 (9.4%)*** |
|  | Non-European region | 1 (0.6%) | - | - | 4 (2.4%) | 5 (2.9%) |
|  | All | 7 (4.1%) | 3 (1.8%) | 8 (4.7%) | 6 (3.5%) | **24 (14.1%)▪** |
| Item 2 | Northern European region | 2 (1.2%) | - | - | - | 2 (1.2%) |
|  | Southern European region | - | - | - | - | - |
|  | English speaking region | 3 (1.8%) | 2 (1.2%) | 5 (2.9%) | 6 (3.5%) | **16 (9.4%)*** |
|  | Non-European region | 1 (0.6%) | - | - | 1 (0.6%) | 2 (1.2%) |
|  | All | 6 (3.5%) | 2 (1.2%) | 5 (2.9%) | 7 (4.1%) | **20 (11.8%)▪** |
| Item 3 | Northern European region | - | - | 1 (0.6%) | - | 1 (0.6%) |
|  | Southern European region | - | - | - | - | - |
|  | English speaking region | 5 (2.9%) | 2 (1.2%) | 2 (1.2%) | 7 (4.1%) | **16 (9.4%)*** |
|  | Non-European region | - | - | - | 1 (0.6%) | 1 (0.6%) |
|  | All | 5 (2.9%) | 2 (1.2%) | 3 (1.8%) | 8 (4.7%) | **18 (10.6%)▪** |
| Item 4 | Northern European region | - | - | - | - | - |
|  | Southern European region | - | - | - | - | - |
|  | English speaking region | 5 (2.9%) | 5 (2.9%) | 1 (0.6%) | 5 (2.9%) | **16 (9.4%)*** |
|  | Non-European region | - | - | - | 2 (1.2%) | 2 (1.2%) |
|  | All | 5 (2.9%) | 5 (2.9%) | 1 (0.6%) | 7 (4.1%) | **18 (10.6%)▪** |
| Item 5 | Northern European region | 2 (1.2%) | 1 (0.6%) | - | - | 3 (1.8%) |
|  | Southern European region | - | - | - | - | - |
|  | English speaking region | 1 (0.6%) | 13 (7.6%) | - | 1 (0.6%) | **15 (8.8%)*** |
|  | Non-European region | - | - | - | 2 (1.2%) | 2 (1.2%) |
|  | All | 3 (1.8%) | 14 (8.2%) | - | 3 (1.8%) | **20 (11.8%)▪** |
| Item 6 | Northern European region | - | - | - | - | - |
|  | Southern European region | - | - | - | - | - |
|  | English speaking region | - | 11 (6.5%) | 3 (1.8%) | 1 (0.6%) | **15 (8.8%)*** |
|  | Non-European region | - | - | - | - | - |
|  | All | - | 11 (6.5%) | 3 (1.8%) | 1 (0.6%) | **15 (8.8%)▪** |
| Item 11 | Northern European region | - | - | - | - | - |
|  | Southern European region | - | - | - | - | - |
|  | English speaking region | 1 (0.6%) | 6 (3.5%) | 7 (4.1%) | 2 (1.2%) | **16 (9.4%)*** |
|  | Non-European region | - | - | - | - | - |
|  | All | 1 (0.6%) | 6 (3.5%) | 7 (4.1%) | 2 (1.2%) | **16 (9.4%)▪** |
| Item 12˜ | Northern European region | 1 (0.6%) | - | - | - | 1 (0.6%) |
|  | Southern European region | - | - | - | - | - |
|  | English speaking region | 2 (1.2%) | 5 (2.9%) | 7 (4.1%) | 2 (1.2%) | **16 (9.4%)*** |
|  | Non-European region | - | - | - | - | - |
|  | All | 3 (1.8%) | 5 (2.9%) | 7 (4.1%) | 2 (1.2%) | **17 (10.0%)▪** |
| Item 14˜ | Northern European region | 1 (0.6%) | 2 (1.2%) | - | - | 3 (1.8%) |
|  | Southern European region | - | - | - | - | - |
|  | English speaking region | 1 (0.6%) | 13 (7.6%) | 1 (0.6%) | 1 (0.6%) | **16 (9.4%)*** |
|  | Non-European region | 1 (0.6%) | 1 (0.6%) | - | - | 2 (1.2%) |
|  | All | 3 (1.8%) | 16 (9.4%) | 1 (0.6%) | 1 (0.6%) | **21 (12.4%)▪** |
| Item 19 | Northern European region | 1 (0.6%) | - | - | - | 1 (0.6%) |
|  | Southern European region | - | - | - | - | - |
|  | English speaking region | 4 (2.4%) | 5 (2.9%) | 6 (3.5%) | 1 (0.6%) | **16 (9.4%)*** |
|  | Non-European region | 1 (0.6%) | - | - | 1 (0.6%) | 2 (1.2%) |
|  | All | 6 (3.5%) | 5 (2.9%) | 6 (3.5%) | 2 (1.2%) | **19 (11.2%)▪** |
| Item 22˜ | Northern European region | 2 (1.2%) | 2 (1.2%) | - | - | 4 (2.4%) |
|  | Southern European region | 2 (1.2%) | - | - | - | 2 (1.2%) |
|  | English speaking region | 5 (2.9%) | 9 (5.3%) | 2 (1.2%) | 2 (1.2%) | **18 (10.6%)*** |
|  | Non-European region | 1 (0.6%) | 3 (1.8%) | 1 (0.6%) | 2 (1.2%) | **7 (4.1%)*** |
|  | All | 10 (5.9%) | 14 (8.2%) | 3 (1.8%) | 4 (2.4%) | **31 (18.2%)▪** |
| Items 23 | Northern European region | 1 (0.6%) | - | - | - | 1 (0.6%) |
|  | Southern European region | - | - | - | - | - |
|  | English speaking region | 8 (4.7%) | 1 (0.6%) | 6 (3.5%) | 1 (0.6%) | **16 (9.4%)*** |
|  | Non-European region | - | - | - | - | - |
|  | All | 9 (5.3%) | 1 (0.6%) | 6 (3.5%) | 1 (0.6%) | **17 (10.0%)▪** |
| Items 24 | Northern European region | 2 (1.2%) | 1 (0.6%) | - | - | 3 (1.8%) |
|  | Southern European region | - | - | - | - | - |
|  | English speaking region | 5 (2.9%) | 3 (1.8%) | 1 (0.6%) | 6 (3.5%) | **15 (8.8%)*** |
|  | Non-European region | - | 1 (0.6%) | - | 1 (0.6%) | 2 (1.2%) |
|  | All | 7 (4.1%) | 5 (2.9%) | 1 (0.6%) | 7 (4.1%) | **20 (11.8%)▪** |
| Items 25 | Northern European region | - | - | - | - | - |
|  | Southern European region | - | - | - | - | - |
|  | English speaking region | 1 (0.6%) | 11 (6.5%) | 2 (1.2%) | 2 (1.2%) | **16 (9.4%)*** |
|  | Non-European region | 1 (0.6%) | 1 (0.6%) | - | - | 2 (1.2%) |
|  | All | 2 (1.2%) | 12 (7.1%) | 2 (1.2%) | 2 (1.2%) | **18 (10.6%)▪** |
| Items 26 | Northern European region | 1 (0.6%) | - | - | - | 1 (0.6%) |
|  | Southern European region | - | - | - | - | - |
|  | English speaking region | 4 (2.4%) | 2 (1.2%) | 2 (1.2%) | 8 (4.7%) | **16 (9.4%)*** |
|  | Non-European region | - | - | - | - | - |
|  | All | 5 (2.9%) | 2 (1.2%) | 2 (1.2%) | 8 (4.7%) | **17 (10.0%)▪** |
| Items 27˜ | Northern European region | 1 (0.6%) | - | 1 (0.6%) | - | 2 (1.2%) |
|  | Southern European region | - | - | - | - | - |
|  | English speaking region | 3 (1.8%) | 3 (1.8%) | 3 (1.8%) | 5 (2.9%) | **14 (8.2%)*** |
|  | Non-European region | 1 (0.6%) | - | - | - | 1 (0.6%) |
|  | All | 5 (2.9%) | 3 (1.8%) | 4 (2.4%) | 5 (2.9%) | **17 (10.0%)▪** |
| Items 29 | Northern European region | - | - | 1 (0.6%) | - | 1 (0.6%) |
|  | Southern European region | - | - | - | - | - |
|  | English speaking region | 6 (3.5%) | 1 (0.6%) | 3 (1.8%) | 6 (3.5%) | **16 (9.4%)*** |
|  | Non-European region | 1 (0.6%) | - | - | - | 1 (0.6%) |
|  | All | 7 (4.1%) | 1 (0.6%) | 4 (2.4%) | 6 (3.5%) | **18 (10.6%)▪** |
| Items 32˜ | Northern European region | 5 (2.9%) | - | - | 1 (0.6%) | 6 (3.5%) |
|  | Southern European region | - | - | - | - | - |
|  | English speaking region | 5 (2.9%) | 4 (2.4%) | 1 (0.6%) | 6 (3.5%) | **16 (9.4%)*** |
|  | Non-European region | 2 (1.2%) | - | - | - | 2 (1.2%) |
|  | All | 12 (7.1%) | 4 (2.4%) | 1 (0.6%) | 7 (4.1%) | **24 (14.1%)▪** |
| Item 33˜ | Northern European region | 2 (1.2%) | 2 (1.2%) | 2 (1.2%) | - | 6 (3.5%) |
|  | Southern European region | - | - | - | - | - |
|  | English speaking region | 3 (1.8%) | 6 (3.5%) | 5 (2.9%) | 1 (0.6%) | **15 (8.8%)*** |
|  | Non-European region | 2 (1.2%) | 1 (0.6%) | - | - | 3 (1.8%) |
|  | All | 7 (4.1%) | 9 (5.3%) | 7 (4.1%) | 1 (0.6%) | **24 (14.1%)▪** |
| Item 38 | Northern European region | 1 (0.6%) | - | - | - | 1 (0.6%) |
|  | Southern European region | - | - | - | - | - |
|  | English speaking region | 5 (2.9%) | 3 (1.8%) | 3 (1.8%) | 5 (2.9%) | **16 (9.4%)*** |
|  | Non-European region | 2 (1.2%) | - | - | - | 2 (1.2%) |
|  | All | 8 (4.7%) | 3 (1.8%) | 3 (1.8%) | 5 (2.9%) | **19 (11.2%)▪** |
| Item 39 | Northern European region | 1 (0.6%) | 1 (0.6%) | 2 (1.2%) | - | 4 (2.4%) |
|  | Southern European region | - | - | - | - | - |
|  | English speaking region | 5 (2.9%) | 2 (1.2%) | 2 (1.2%) | 7 (4.1%) | **16 (9.4%)*** |
|  | Non-European region | - | - | - | - | - |
|  | All | 6 (3.5%) | 3 (1.8%) | 4 (2.4%) | 7 (4.1%) | **20 (11.8%)▪** |
| Item 44˜ | Northern European region | - | - | - | - | - |
|  | Southern European region | - | - | - | - | - |
|  | English speaking region | 2 (1.2%) | - | 1 (0.6%) | 2 (1.2%) | 5 (2.9%) |
|  | Non-European region | 3 (1.8%) | 2 (1.2%) | - | - | 5 (2.9%) |
|  | All | 5 (2.9%) | 2 (1.2%) | 1 (0.6%) | 2 (1.2%) | **10 (5.9%)▪** |
| Item 45 | Northern European region | - | - | 1 (0.6%) | - | 1 (0.6%) |
|  | Southern European region | - | - | - | - | - |
|  | English speaking region | 2 (1.2%) | - | - | 1 (0.6%) | 3 (1.8%) |
|  | Non-European region | - | - | - | - | - |
|  | All | 2 (1.2%) | - | 1 (0.6%) | 1 (0.6%) | 4 (2.4%) |
| Item 46˜ | Northern European region | 3 (1.8%) | - | - | 1 (0.6%) | 4 (2.4%) |
|  | Southern European region | - | - | - | - | - |
|  | English speaking region | - | - | - | - | - |
|  | Non-European region | - | 1 (0.6%) | - | 1 (0.6%) | 2 (1.2%) |
|  | All | 3 (1.8%) | 1 (0.6%) | - | 2 (1.2%) | 6 (3.5%) |
| Item 47˜ | Northern European region | - | - | - | 1 (0.6%) | 1 (0.6%) |
|  | Southern European region | - | - | - | - | - |
|  | English speaking region | - | - | - | 1 (0.6%) | 1 (0.6%) |
|  | Non-European region | - | - | - | 1 (0.6%) | 1 (0.6%) |
|  | All | - | - | - | 3 (1.8%) | 3 (1.8%) |
| Item 48 | Northern European region | - | 3 (1.8%) | - | - | 3 (1.8%) |
|  | Southern European region | - | - | - | - | - |
|  | English speaking region | - | - | - | - | - |
|  | Non-European region | - | - | - | - | - |
|  | All | - | 3 (1.8%) | - | - | 3 (1.8%) |
| Item 50 | Northern European region | - | - | - | - | - |
|  | Southern European region | - | - | - | - | - |
|  | English speaking region | - | - | - | - | - |
|  | Non-European region | - | - | - | - | - |
|  | All | - | - | - | - | - |
| Item 51˜ | Northern European region | 29 (17.1%) | 20 (11.8%) | 2 (1.2%) | - | **51 (30.0%)*** |
|  | Southern European region | - | - | - | - | - |
|  | English speaking region | - | - | - | - | - |
|  | Non-European region | - | - | - | - | - |
|  | All | 29 (17.1%) | 20 (11.8%) | 2 (1.2%) | - | **51 (30.0%)▪** |
| Item 54˜ | Northern European region | - | 10 (5.9%) | - | - | **10 (5.9%)*** |
|  | Southern European region | - | - | - | - | - |
|  | English speaking region | - | - | - | - | - |
|  | Non-European region | - | 3 (1.8%) | 1 (0.6%) | - | 4 (2.4%) |
|  | All | - | 13 (7.6%) | 1 (0.6%) |  | **14 (8.2%)▪** |
| Item 55 | Northern European region | 1 (0.6%) | 1 (0.6%) | - | - | 2 (1.2%) |
|  | Southern European region | - | - | - | - | - |
|  | English speaking region | - | - | - | - | - |
|  | Non-European region | - | - | - | - | - |
|  | All | 1 (0.6%) | 1 (0.6%) | - | - | 2 (1.2%) |
| Item 56 | Northern European region | 1 (0.6%) | 2 (1.2%) | - | - | 3 (1.8%) |
|  | Southern European region | - | - | - | - | - |
|  | English speaking region | - | - | - | - | - |
|  | Non-European region | - | - | - | - | - |
|  | All | 1 (0.6%) | 2 (1.2%) | - | - | 3 (1.8%) |
| Item 59 | Northern European region | 1 (0.6%) | - | - | - | 1 (0.6%) |
|  | Southern European region | - | - | - | - | - |
|  | English speaking region | - | - | - | - | - |
|  | Non-European region | - | - | - | - | - |
|  | All | 1 (0.6%) | - | - | - | 1 (0.6%) |
| Supplemental File 3; Table 1. Acceptability of participant data (N=170). *= >5 participants per region and ▪= >5% participants (8.5 participants) (consider for revision), ˜= item ≥2 dyads raised same concern, accordingly rephrased. | | | | | | |

| Item | N | Mean difference  [patient - proxy score] | Standard deviation |
| --- | --- | --- | --- |
| IADL Q1 | 77 | -0.01 | 0.88 |
| Cognitively impaired | 34 | -0.12 | 1.09 |
| Cognitively unimpaired | 39 | 0.08 | 0.70 |
| IADL Q2 | 77 | -0.18 | 0.76 |
| Cognitively impaired | 35 | -0.20 | 0.96 |
| Cognitively unimpaired | 38 | -0.13 | 0.53 |
| IADL Q3 | 74 | -0.11 | 1.05 |
| Cognitively impaired | 31 | -0.29 | 1.24 |
| Cognitively unimpaired | 41 | 0.02 | 0.91 |
| IADL Q4 | 71 | -0.18 | 0.89 |
| Cognitively impaired | 29 | -0.24 | 1.27 |
| Cognitively unimpaired | 38 | -0.11 | 0.51 |
| IADL Q5 | 68 | -0.06 | 0.94 |
| Cognitively impaired | 29 | -0.24 | 1.27 |
| Cognitively unimpaired | 37 | 0.11 | 0.81 |
| IADL Q6 | 67 | -0.10 | 0.82 |
| Cognitively impaired | 25 | -0.36 | 0.95 |
| Cognitively unimpaired | 39 | 0.05 | 0.72 |
| IADL Q11 | 82 | -0.10 | 0.87 |
| Cognitively impaired | 35 | -0.29 | 1.07 |
| Cognitively unimpaired | 43 | 0.02 | 0.67 |
| IADL Q12 | 71 | 0.00 | 0.88 |
| Cognitively impaired | 26 | -0.27 | 1.08 |
| Cognitively unimpaired | 42 | 0.10 | 0.66 |
| IADL Q14 | 32 | 0.00 | 1.05 |
| Cognitively impaired | 13 | -0.38 | 1.45 |
| Cognitively unimpaired | 17 | 0.24 | 0.56 |
| IADL Q19 | 79 | -0.10 | 0.90 |
| Cognitively impaired | 35 | -0.29 | 1.13 |
| Cognitively unimpaired | 40 | 0.00 | 0.60 |
| IADL Q22 | 81 | -0.16 | 1.01 |
| Cognitively impaired | 36 | -0.33 | 1.35 |
| Cognitively unimpaired | 41 | -0.05 | 0.59 |
| IADL Q23 | 62 | -0.05 | 1.01 |
| Cognitively impaired | 22 | -0.14 | 1.32 |
| Cognitively unimpaired | 36 | 0.00 | 0.79 |
| IADL Q24 | 78 | -0.23 | 0.93 |
| Cognitively impaired | 31 | -0.55 | 1.12 |
| Cognitively unimpaired | 43 | 0.00 | 0.72 |
| IADL Q25 | 82 | 0.04 | 0.92 |
| Cognitively impaired | 35 | -0.20 | 1.11 |
| Cognitively unimpaired | 43 | 0.23 | 0.72 |
| IADL Q26 | 84 | -0.11 | 0.84 |
| Cognitively impaired | 36 | -0.14 | 0.99 |
| Cognitively unimpaired | 44 | -0.09 | 0.74 |
| IADL Q27 | 77 | 0.26 | 0.86 |
| Cognitively impaired | 32 | 0.19 | 1.00 |
| Cognitively unimpaired | 41 | 0.29 | 0.75 |
| IADL Q29 | 78 | -0.08 | 0.98 |
| Cognitively impaired | 31 | -0.39 | 1.17 |
| Cognitively unimpaired | 43 | 0.21 | 0.71 |
| IADL Q32 | 71 | 0.01 | 0.93 |
| Cognitively impaired | 28 | -0.11 | 1.32 |
| Cognitively unimpaired | 40 | 0.10 | 0.59 |
| IADL Q33 | 66 | -0.27 | 0.90 |
| Cognitively impaired | 25 | -0.60 | 1.08 |
| Cognitively unimpaired | 39 | -0.08 | 0.74 |
| IADL Q38 | 77 | 0.13 | 0.94 |
| Cognitively impaired | 34 | 0.24 | 1.08 |
| Cognitively unimpaired | 39 | -0.08 | 0.62 |
| IADL Q39 | 72 | -0.08 | 1.02 |
| Cognitively impaired | 29 | -0.28 | 1.16 |
| Cognitively unimpaired | 40 | 0.00 | 0.88 |
| IADL Q44 | 83 | -0.17 | 0.92 |
| Cognitively impaired | 36 | -0.42 | 1.03 |
| Cognitively unimpaired | 43 | 0.00 | 0.82 |
| IADL Q45 | 77 | -0.12 | 0.83 |
| Cognitively impaired | 34 | -0.32 | 0.98 |
| Cognitively unimpaired | 39 | 0.05 | 0.69 |
| IADL Q46 | 83 | -0.25 | 0.91 |
| Cognitively impaired | 37 | -0.57 | 1.09 |
| Cognitively unimpaired | 43 | -0.05 | 0.58 |
| IADL Q47 | 63 | -0.16 | 0.88 |
| Cognitively impaired | 30 | -0.23 | 1.17 |
| Cognitively unimpaired | 31 | -0.06 | 0.51 |
| IADL Q48 | 76 | -0.20 | 1.03 |
| Cognitively impaired | 31 | -0.48 | 1.34 |
| Cognitively unimpaired | 41 | 0.00 | 0.71 |
| IADL Q50 | 58 | -0.24 | 1.03 |
| Cognitively impaired | 23 | -0.70 | 1.26 |
| Cognitively unimpaired | 32 | 0.09 | 0.73 |
| IADL Q51 | 77 | -0.12 | 0.89 |
| Cognitively impaired | 33 | -0.21 | 0.99 |
| Cognitively unimpaired | 40 | -0.05 | 0.85 |
| IADL Q54 | 76 | -0.01 | 0.89 |
| Cognitively impaired | 30 | -0.20 | 1.06 |
| Cognitively unimpaired | 42 | 0.12 | 0.74 |
| IADL Q55 | 77 | -0.17 | 0.92 |
| Cognitively impaired | 31 | -0.48 | 1.09 |
| Cognitively unimpaired | 42 | 0.02 | 0.75 |
| IADL Q56 | 77 | -0.04 | 0.97 |
| Cognitively impaired | 31 | -0.23 | 1.20 |
| Cognitively unimpaired | 42 | 0.07 | 0.78 |
| IADL Q59 | 81 | -0.11 | 1.03 |
| Cognitively impaired | 37 | -0.19 | 1.24 |
| Cognitively unimpaired | 41 | -0.05 | 0.81 |
| Supplemental File 3; Table 2. Congruency; mean differences between patient score and proxy score per item. | | | |

| Dyads | Raw agreement | Cohen’s Kappa (κ) | Classification^27^ | Cognitive status |
| --- | --- | --- | --- | --- |
| Dyad 1 | 45,2% | -0.16 | Disagreement | Not cognitively impaired |
| Dyad 2 | 60,0% | -0.14 | Disagreement | Not cognitively impaired |
| Dyad 3 | 17,4% | -0.11 | Disagreement | Not cognitively impaired |
| Dyad 4 | 67,9% | -0.06 | Disagreement | Not cognitively impaired |
| Dyad 5 | 74,2% | -0.06 | Disagreement | Not cognitively impaired |
| Dyad 6 | 92,9% | -0.02 | Disagreement | Not cognitively impaired |
| Dyad 7 | 30,4% | -0.01 | Disagreement | Not cognitively impaired |
| Dyad 8 | 36,0% | -0.00 | Disagreement | Not cognitively impaired |
| Dyad 9 | 71,4% | -0.00 | Disagreement | Not cognitively impaired |
| Dyad 10 | 41,9% | 0.01 | Slight agreement | Not cognitively impaired |
| Dyad 11 | 31,0% | 0.07 | Slight agreement | Not cognitively impaired |
| Dyad 12 | 56,7% | 0.13 | Slight agreement | Not cognitively impaired |
| Dyad 13 | 41,4% | 0.17 | Slight agreement | Not cognitively impaired |
| Dyad 14 | 42,9% | 0.17 | Slight agreement | Not cognitively impaired |
| Dyad 15 | 43,3% | 0.18 | Slight agreement | Not cognitively impaired |
| Dyad 16 | 43,8% | 0.20 | Slight agreement | Not cognitively impaired |
| Dyad 17 | 53,6% | 0.21 | Fair agreement | Not cognitively impaired |
| Dyad 18 | 84,4% | 0.22 | Fair agreement | Not cognitively impaired |
| Dyad 19 | 46,7% | 0.24 | Fair agreement | Not cognitively impaired |
| Dyad 20 | 50,0% | 0.24 | Fair agreement | Not cognitively impaired |
| Dyad 21 | 60,7% | 0.25 | Fair agreement | Not cognitively impaired |
| Dyad 22 | 87,5% | 0.26 | Fair agreement | Not cognitively impaired |
| Dyad 23 | 67,7% | 0.27 | Fair agreement | Not cognitively impaired |
| Dyad 24 | 60,0% | 0.27 | Fair agreement | Not cognitively impaired |
| Dyad 25 | 69,0% | 0.28 | Fair agreement | Not cognitively impaired |
| Dyad 26 | 58,1% | 0.31 | Fair agreement | Not cognitively impaired |
| Dyad 27 | 45,0% | 0.32 | Fair agreement | Not cognitively impaired |
| Dyad 28 | 63,0% | 0.33 | Fair agreement | Not cognitively impaired |
| Dyad 29 | 56,3% | 0.34 | Fair agreement | Not cognitively impaired |
| Dyad 30 | 65,5% | 0.34 | Fair agreement | Not cognitively impaired |
| Dyad 31 | 82,8% | 0.34 | Fair agreement | Not cognitively impaired |
| Dyad 32 | 61,3% | 0.40 | Fair agreement | Not cognitively impaired |
| Dyad 33 | 59,1% | 0.45 | Moderate agreement | Not cognitively impaired |
| Dyad 34 | 64,3% | 0.50 | Moderate agreement | Not cognitively impaired |
| Dyad 35 | 100,0% | 1.00 | Perfect agreement | Not cognitively impaired |
| Dyad 36 | 100,0% | 1.00 | Perfect agreement | Not cognitively impaired |
| Dyad 37 | 76,7% | # | Indetermined | Not cognitively impaired |
| Dyad 38 | 80,6% | # | Indetermined | Not cognitively impaired |
| Dyad 39 | 83,9% | # | Indetermined | Not cognitively impaired |
| Dyad 40 | 92,9% | # | Indetermined | Not cognitively impaired |
| Dyad 41 | 96,7% | # | Indetermined | Not cognitively impaired |
| Dyad 42 | 96,9% | # | Indetermined | Not cognitively impaired |
| Dyad 43 | 100,0% | * | Indetermined | Not cognitively impaired |
| Dyad 44 | 100,0% | * | Indetermined | Not cognitively impaired |
|  |  |  |  |  |
| Dyad 45 | 12,9% | -0.16 | Disagreement | Cognitively impaired |
| Dyad 46 | 33,3% | -0.14 | Disagreement | Cognitively impaired |
| Dyad 47 | 40,7% | -0.09 | Disagreement | Cognitively impaired |
| Dyad 48 | 19,4% | -0.08 | Disagreement | Cognitively impaired |
| Dyad 49 | 50,0% | -0.06 | Disagreement | Cognitively impaired |
| Dyad 50 | 90,9% | -0.05 | Disagreement | Cognitively impaired |
| Dyad 51 | 23,8% | -0.04 | Disagreement | Cognitively impaired |
| Dyad 52 | 12,9% | -0.02 | Disagreement | Cognitively impaired |
| Dyad 53 | 6,7% | -0.00 | Disagreement | Cognitively impaired |
| Dyad 54 | 32,1% | 0.00 | Slight agreement | Cognitively impaired |
| Dyad 55 | 26,7% | 0.04 | Slight agreement | Cognitively impaired |
| Dyad 56 | 41,4% | 0.05 | Slight agreement | Cognitively impaired |
| Dyad 57 | 72,0% | 0.05 | Slight agreement | Cognitively impaired |
| Dyad 58 | 67,7% | 0.06 | Slight agreement | Cognitively impaired |
| Dyad 59 | 18,8% | 0.07 | Slight agreement | Cognitively impaired |
| Dyad 60 | 37,0% | 0.07 | Slight agreement | Cognitively impaired |
| Dyad 61 | 33,3% | 0.08 | Slight agreement | Cognitively impaired |
| Dyad 62 | 51,9% | 0.08 | Slight agreement | Cognitively impaired |
| Dyad 63 | 34,5% | 0.09 | Slight agreement | Cognitively impaired |
| Dyad 64 | 62,1% | 0.12 | Slight agreement | Cognitively impaired |
| Dyad 65 | 44,8% | 0.12 | Slight agreement | Cognitively impaired |
| Dyad 66 | 64,3% | 0.15 | Slight agreement | Cognitively impaired |
| Dyad 67 | 37,5% | 0.16 | Slight agreement | Cognitively impaired |
| Dyad 68 | 30,8% | 0.16 | Slight agreement | Cognitively impaired |
| Dyad 69 | 53,3% | 0.20 | Slight agreement | Cognitively impaired |
| Dyad 70 | 42,3% | 0.21 | Fair agreement | Cognitively impaired |
| Dyad 71 | 42,1% | 0.22 | Fair agreement | Cognitively impaired |
| Dyad 72 | 47,8% | 0.25 | Fair agreement | Cognitively impaired |
| Dyad 73 | 85,2% | 0.26 | Fair agreement | Cognitively impaired |
| Dyad 74 | 51,6% | 0.26 | Fair agreement | Cognitively impaired |
| Dyad 75 | 50,0% | 0.32 | Fair agreement | Cognitively impaired |
| Dyad 76 | 69,2% | 0.34 | Fair agreement | Cognitively impaired |
| Dyad 77 | 75,0% | 0.52 | Moderate agreement | Cognitively impaired |
| Dyad 78 | 100,0% | 1.00 | Perfect agreement | Cognitively impaired |
| Dyad 79 | 0,0% | # | Indetermined | Cognitively impaired |
| Dyad 80 | 13,8% | # | Indetermined | Cognitively impaired |
| Dyad 81 | 100,0% | * | Indetermined | Cognitively impaired |
|  |  |  |  |  |
| Dyad 82 | 36,0% | 0.17 | Slight agreement | No cognitive data available |
| Dyad 83 | 60,0% | 0.37 | Fair agreement | No cognitive data available |
| Dyad 84 | 91,3% | 0.87 | Sustantial agreement | No cognitive data available |
| Dyad 85 | 96,7% | # | Indetermined | No cognitive data available |
| Supplemental File 3; Table 3. Congruency between patient and proxy agreement. *=Kappa could not be computed because both patient and proxy had no rating variation. #= Kappa could not be computed because either the patient or the proxy had no rating variation. | | | | |
